# Effects of Vitamin A on In Vitro Maturation of Pre-Pubertal Mouse Spermatogonial Stem Cells

**DOI:** 10.1371/journal.pone.0082819

**Published:** 2013-12-09

**Authors:** Albanne Travers, Brahim Arkoun, Athmane Safsaf, Jean-Pierre Milazzo, Anne Absyte, Amandine Bironneau, Anne Perdrix, Louis Sibert, Bertrand Macé, Bruno Cauliez, Nathalie Rives

**Affiliations:** 1 EA 4308 "Gametogenesis and Gamete Quality", Reproductive Biology Laboratory - CECOS, Rouen University Hospital, Institute for Biomedical Research, University of Rouen, Rouen, France; 2 EA 4308 "Gametogenesis and Gamete Quality", Department of Urology, Rouen University Hospital, Institute for Biomedical Research, University of Rouen, Rouen, France; 3 Biochemistry Laboratory, Rouen University Hospital, Institute for Biomedical Research, University of Rouen, Rouen, France; University Hospital of Münster, Germany

## Abstract

Testicular tissue cryopreservation is the only potential option for fertility preservation in pre-pubertal boys exposed to gonadotoxic treatment. Completion of spermatogenesis after *in vitro* maturation is one of the future uses of harvested testicular tissue. The purpose of the current study was to evaluate the effects of vitamin A on in vitro maturation of fresh and frozen-thawed mouse pre-pubertal spermatogonial stem cells in an organ culture system. Pre-pubertal CD1 mouse fresh testes were cultured for 7 (D7), 9 (D9) and 11 (D11) days using an organ culture system. Basal medium was supplemented with different concentrations of retinol (Re) or retinoic acid (RA) alone or in combination. Seminiferous tubule morphology (tubule diameter, intra-tubular cell type), intra-tubular cell death and proliferation (PCNA antibody) and testosterone level were assessed at D7, D9 and D11. Pre-pubertal mouse testicular tissue were frozen after a soaking temperature performed at -7°C, -8°C or -9°C and after thawing, were cultured for 9 days, using the culture medium preserving the best fresh tissue functionality. Retinoic acid at 10^-6^M and retinol at 3.3.10^-7^M, as well as retinol 10^-6^M are favourable for seminiferous tubule growth, maintenance of intra-tubular cell proliferation and germ cell differentiation of fresh pre-pubertal mouse spermatogonia. Structural and functional integrity of frozen-thawed testicular tissue appeared to be well-preserved after soaking temperature at -8°C, after 9 days of organotypic culture using 10^-6^M retinol. RA and Re can control in vitro germ cell proliferation and differentiation. Re at a concentration of 10^-6^M maintains intra-tubular cell proliferation and the ability of spermatogonia to initiate spermatogenesis in fresh and frozen pre-pubertal mouse testicular tissue using a soaking temperature at -8°C. Our data suggested a possible human application for in vitro maturation of cryopreserved pre-pubertal testicular tissue.

## Introduction

Spermatogenesis is a highly organized process of cell proliferation and terminal differentiation that leads to the formation of mature spermatozoa. Several external factors are susceptible to impair spermatogenesis and more specifically spermatogonial stem cells, such as cancer treatment, chemotherapy or radiotherapy, with possible transient or permanent spermatogenesis arrest [1].

Gonad damage is a relatively common consequence of cancer treatment. Indeed, 10 to 100% of cured patients will show semen parameter alterations after treatment and an average of 15 to 30% of them remain infertile in the long term [2,3]. Since several years, sperm cryopreservation is proposed for young adult males and adolescents prior to gonadotoxic treatment [4]. However, for pre-pubertal boys exposed to gonadotoxic treatment, testicular tissue freezing appears to be the only potential option to preserve their future fertility, even if this procedure remains actually not currently proposed. However, clinical experience has been reported [5-7]. Open testicular biopsy is generally carried out under general anaesthesia in combination with another clinical care of the patient (tumor ablation, central line placement) [5-7]. The parents of young boys consented to testicular biopsy in 76% [5] or 93.5% [7] of cases and few sequelae occurred during intra- or post-operative procedure [5,7].

Testicular tissue freezing offers the possibility to preserve the different testicular cells, to maintain spermatogonial stem cells into their specific environment, the so-called "niche", and to respect the interactions between germ cells and Sertoli cells [8,9]. Several potential options can be proposed to restore fertility using frozen testicular tissue. Spermatogenesis can be completed after in vitro maturation, germ cell transplantation, xenologous or autologous testis fragment grafting [3,10,11]. However, these approaches may present some limitation. Indeed, in case of leukaemia or any other metastatic childhood cancer, malignant cell contamination of harvested testicular tissue may exist. In this case, germ cell transplantation or autologous testicular fragment grafting may induce a malignant relapse in the transplanted patient [12]. The use of a xenogenic intermediate host could be an alternative option to avoid the transfer of malignant cells to the patient. Some safety issues should be evaluated and more particularly the risk of zoonosis, because spermatozoa obtain after xenografting would be used for fertilization [13]. As a consequence, in vitro differentiation of spermatogonial stem cells could be an option to restore fertility and to avoid malignant relapse and risk of zoonosis.

The whole process of spermatogenesis requires endocrine and local paracrine regulation and direct cell to cell interactions (between peritubular cells and intra-tubular cells, germ cells and germ cells, Sertoli cells and germ cells) [14]. Therefore, organ culture experiments appear to be of great interest to reproduce in vitro spermatogenesis because they preserve testicular tissue architecture and intercellular communication [15]. Many groups have focused their studies on male germ cell in vitro differentiation, mostly using tissue or organ culture [16]. Organotypic culture system has proven its efficiency in some animal models like in rat and mouse foetal and neonatal testis [17] as well as in human foetal testis [18]. In rodents, foetal and neonatal Leydig cells, Sertoli cells and germ cells can develop normally over a period of 1-2 weeks in an organotypic culture system according to a pattern similar to the pattern observed in vivo. Furthermore, the level of differentiated functions (for example, gonocyte mitosis and differentiation into spermatogonia, seminiferous cord organization, AMH and testosterone production) does not differ from in vivo conditions and demonstrate the potential usefulness of organotypic culture system to explore the effects of several factors (age, time, dose-dependent hormone effects, paracrine and autocrine factors, environmental factors...) [17]. After two weeks of human foetal testis organotypic culture, all the germ cell types remained detectable and some of them were still able to divide and to differentiate with a rate comparable as observed in vivo. Thus, a good maintenance of the general architecture of the testis was confirmed suggesting, as for rodents, that this culture system represent a valuable tool for testing the effects of biological and chemical agents on in vitro maturation of testicular tissue [18]. 

In addition to playing a fundamental role in several biological processes such as vision, growth and differentiation of numerous cell types, retinoids (retinol [RE] and retinoic acid [RA]) are clearly involved in the regulation of testicular functions by acting directly on the three main testicular cell types (Sertoli cells, germ cells and Leydig cells) [19]. Indeed, retinoids control several functions in adult Sertoli cells e.g. stimulation of secretion (transferrin, Androgen Binding Protein), signalling pathway regulation (expression of androgen receptor, production of cAMP in response to Follicule Stimulating Hormone [FSH]), metabolism modulation (for example, ornithine decarboxylase and cytochrome c oxidase expression) [20]. Furthermore, retinoids are precocious and transient regulators of testosterone secretion in rodents [21,22] and in humans [18]. Retinoids play also a major role during the proliferation and differentiation of type A spermatogonia, the transition from round to elongated spermatids and spermiation [20]. Indeed, in adults, vitamin A deficiency leads to spermatogenesis arrest [23,24] and in testes of vitamin-A deficient rodents, retinoid supplementation retriggered male gametogenesis up to the spermatocyte stage that occurred, in a synchronous manner, in all seminiferous tubules [25-27]. 

Since RE and RA are clearly involved in spermatogenesis regulation, the first aim of our study was to evaluate the effect of different concentrations of these two retinoids on in vitro proliferation and differentiation of fresh pre-pubertal murine spermatogonial stem cells in an organ culture system. Leydig cell functional integrity was checked by assessing in vitro steroidogenesis. Then, in order to improve the freezing protocol of pre-pubertal mouse testicular tissue, three different soaking temperatures were tested and the most favourable culture condition was used to assess the functionality of frozen-thawed mouse testicular tissue in the different freezing conditions. The other freezing protocol parameters have been assessed previously [9,28,29].

## Materials and Methods

### Mice and testis collection

All experimental procedures were approved by the Institutional Animal Care and Use Committee of Rouen Medical University under licence number 76-120. Six to seven day-old CD-1 mice were euthanised by decapitation (Charles River Breeding Laboratories, L'Arbresle, France). The testes were excised and rinsed in Dulbecco modified Eagle medium F12 (DMEM/F12) (Sigma-Aldrich, Saint-Quentin Fallavier, France), with 10% (v/v) of foetal calf serum (FCS) (Eurobio, Courtaboeuf, France). The tunica albuginea was removed and testes were rinsed in the same medium as mentioned above and maintained at 4°C until use. The testes were transferred either into a culture medium drop, if cultured, or into cryovial, if frozen. For each culture condition tested, six testes from mice of different litters were cultured for 7 (D7), 9 (D9) and 11 (D11) days. The testes of 7-, 14-, 16- and 18-day-old mice, corresponding respectively to Day 0 (D0) and the three different culture times (D7, D9 and D11 respectively), were used as in vivo controls and were fixed in Bouin solution (HT10132, Sigma-Aldrich).

### Organ Culture System

The basal culture medium was composed of DMEM/F12 gentamycin (25µg/mL), streptomycin (50mg/L), penicillin (39UI/mL), pyruvate (10^-3^M), insulin (10mg/L), Apo-transferrin (10mg/L) (Sigma-Aldrich) supplemented with Luteinising Hormone [LH] (10UI/L) (Gonadotrophine Chorionique Endo^®^, Organon Schering Plough, Courbevoie, France), FSH (50UI/L) (Puregon^®^, Organon Schering Plough), vitamin C (10^-4^M) and vitamin E (2.10^-2^M) (Sigma-Aldrich).

 Several Re (retinol^®^, Sigma-Aldrich) and RA (retinoic acid^®^, Sigma-Aldrich) concentrations were evaluated separately or in combination for each culture time. Eleven culture media were compared: (i) basal medium without RA or Re [T], (ii) 3.3.10^-7^M RA and 3.3.10^-7^M Re [RARE], (iii) 3.3.10^-7^M RA and 10^-6^M Re [RARE6], (iv) 3.3.10^-7^M RA and 10^-5^M Re [RARE5], (v) 3.3.10^-7^M Re and 10^-6^M RA [RERA6], (vi) 3.3.10^-7^M Re and 10^-5^M RA [RERA5], (vii) 10^-6^M RA [RA6], (viii) 10^-6^M Re [RE6], (ix) 10^-5^M Re [RE5], (x) 10^-4^M Re [RE4] and (xi) 10^-3^M Re [RE3].

 Testes were cut into 25 pieces of approximately 0.1mm^3^ (0.3mm x 0.5mm x 1mm of size). All the pieces from the same testis were cultured for 7, 9 or 11 days at 32°C in a humidified atmosphere containing 95% air and 5% CO_2_, on Millicell-HA membrane (pore size 0.45µm; Millipore, Carrigtwohill, Co. Cork, Ireland) in tissue culture dishes (Falcon 353037^®^, Becton Dickinson, Le Pont de Claix, France) containing 0.4mL of culture medium. The culture media were changed every two days for each culture condition tested and stored at -20°C until testosterone level measurements. Culture was stopped by adding Bouin solution.

### Cryopreservation

The testes were individually placed into cryovials (Cryotube^™^ Vials, Nunc, Roskilde, Denmark) containing 1.5mL of cryoprotective medium consisted of Leibovitz^®^ L-15 medium (Eurobio) supplemented with 1.5M dimethylsulphoxide (DMSO, Sigma-Aldrich), 0.05M sucrose (Sigma-Aldrich) and 10% (v/v) FCS (Eurobio), and were equilibrated at 4°C for 30 minutes. The testes were frozen in a programmable Minicool 40PC freezer (Air Liquide, Paris, France) with a controlled slow freezing protocol: start at 5°C, 2°C/min until temperature stabilisation phase for 8 minutes assessed at -7°C, -8°C or -9°C, 0.3°C/min to -40°C, 25°C/min to -150°C. After the freezing procedure, the samples were plunged and stored into liquid nitrogen at -196°C.

Testicular tissue thawing was performed after several baths with a progressive dilution of cryoprotectant as previously described [9]. Briefly, samples were thawed rapidly in a 37°C water bath and cryomedium was removed in a four-stage procedure with decreased concentration of cryoprotectant.

### Identification of Sertoli cells and germ cells

Tissue sections (3 µm) were stained for Tra98 (for spermatogonia as well as leptotene/zygotene and pachytene spermatocytes I). Then, serial tissue sections were stained with promyelocytic leukemia Zinc Finger (Plzf) or c-kit to identify undifferentiated or differentiated spermatogonia respectively.

Tissue sections were deparaffinised, rehydrated and incubated in an antigen-retrieval solution (10mM citrate, pH6) for 40 minutes at 96°C. All incubation steps were performed at room temperature (except for primary antibody) and followed by wash with TBST (1:10; Tris Buffered Saline Tween 20 10x concentrate, Dako, Trappes, France). Endogenous peroxidases were inactivated by a 5 minute treatments with Peroxidase Blocking Solution (Dako) before overnight incubation at 4°C with the primary antibody [rat anti-Tra98 antibody (1:50; JLP antibody, Abcam, Paris, France), mouse Promyelocytic Leukemia Zinc Finger (Plzf) antibody (1:100; Santa Cruz Biotechnology, Heidelberg, Germany) and goat c-kit antibody (1:100; Santa Cruz Biotechnology, Heidelberg, Germany)].

For the detection of Tra98, sections were incubated with a secondary antibody (1:200; Polyclonal Rabbit anti-rat Immunoglobulins/HRP, Dako), and subsequently incubated with biotinylated tertiary antibody (1:200; Polyclonal Swine anti-rabbit Immunoglobulins/Biotinylated, Dako). Plzf and c-kit were detected using biotinylated rabbit anti-mouse IgG (1:200; kit ultravision plus HRP TP-060-HLX, Microm Microtech, Francheville, France) and donkey anti-goat IgG (1:100; Santa Cruz Biotechnology) respectively. Specific staining was achieved by incubation with a peroxidase-conjugated streptavidin (Dako) for 15 minutes, then, with a 3,3'-diaminobenzidine substrate for 1-3 min. Negative controls were performed by omitting primary antibody. The nuclei were counterstained with haematoxylin (Dako) and 30 cross-sectioned tubules were examined at ×500 magnification under a light microscope (DM4000B, Leica, Solms, Germany) equipped with Leica Application Suite software (LAS Core v2.4, Leica). If more than one section was evaluated to obtain 30 cross-sectioned tubules, the distance between two consecutive sections was 10µm. A cross-sectioned tubule was defined when a ratio of less than 1.5 was observed between the longest diameter of the tubule and the diameter perpendicular to it. The seminiferous tubule area as well as the number and the type of intra-tubular cells were assessed for each culture condition.

Rat Tra98 monoclonal antibody recognizes specific mouse germ cell nuclear antigen [30] and allows accurate distinction between Sertoli cells (Tra98-negative cells) with irregular blue nuclei and germ cells (Tra98-positive cells) with brown nuclei. Then, the different types of germ cells were classified as spermatogonia (smooth spherical brown nuclei), leptotene/zygotene primary spermatocytes (irregular spherical brown nuclei with condensed chromatin) or pachytene primary spermatocytes (irregular spherical brown nuclei with highly condensed chromatin). The number of residual Sertoli cells and spermatogonia was assessed to obtain a ratio between Sertoli cells and spermatogonia in seminiferous tubules over time and was compared to in vivo condition.

### Quantification of proliferating Sertoli cells and germ cells

Two serial sections of paraffin embedded tissue (3µm of thickness) were used to determine the proliferation rate of Sertoli cells and germ cells. The first serial section was processed for Proliferating Cell Nuclear Antigen antibody (PCNA) used as a marker of cell proliferative ability, according to the manufacturer's instructions (Invitrogen, Camarillo, CA, USA). Tra98 immunostaining, as described above, was applied to the second serial section. After substrate reaction, cells were counterstained with haematoxylin (Dako) and analysed under a light microscope (DM4000B, Leica) at ×500 magnification. For each culture condition, germ cells recognized by Tra98 (Tra98-positive cells) were identified in section processed for PCNA immunostaining. In this way, the percentages of PCNA positive germ cells and PCNA positive Sertoli cells (with brown nuclei, respectively detected or not by Tra98 monoclonal antibody) were determined after examination of at least 500 intra-tubular cells. Negative controls were performed by omitting the primary antibody. If more than one section was evaluated to obtain 500 intra-tubular cells, the distance between two consecutive serial sections was 10µm.

### Cellular Death Assessment

Cellular death via necrosis was evaluated using pyknotic nuclei quantification in seminiferous tubules. Pyknotic nuclei assessment was carried out in 30 cross-sectioned tubules stained with Tra98 antibody (see above). Briefly, seminiferous tubules were classified as no pyknotic (<10% of pyknotic nuclei per seminiferous tubules), partially (from 10 to 90% of pyknotic nuclei per seminiferous tubules) or totally pyknotic (>90% of pyknotic nuclei per seminiferous tubules) and the number of pyknotic nuclei was also recorded in totally and partially necrotic seminiferous tubules. A pyknotic nucleus was defined as spherical, shrink with highly condensed chromatin.

 Testosterone measurement using radioimmunology assay In vitro steroidogenesis was evaluated by measuring testosterone secretion into the medium. Media samples were stored at -20°C after 3, 5, 7, 9 and 11 days of culture and were analysed using a direct radioimmunoassay (testosterone kit (IM1087); Immunotech Beckman-Coulter Company, Roissy, France). The samples were assayed in duplicate according to manufacturer’s recommendations. The assay had a lower limit of sensitivity of 0.04 ng/mL with average intra- and inter-assay variations of 7.2 and 10.7% of respectively.

### Statistical analysis

Statistical analysis was performed for all experiments using the Friedman test (for global comparison between all conditions), the Wilcoxon test for paired rank comparisons or the Mann-Whitney test for unpaired rank comparisons. The data are presented as the means ± SEM. A p-value below 0.05 was considered statistically significant.

## Results

### Organ culture of fresh pre-pubertal mice testicular tissue

#### Seminiferous tubule growth (Figure 1A_1_ to 1A_3_ and Figure 2)

The seminiferous tubule area increased significantly during the culture regardless of the concentrations of Re and RA used (p<0.01), except for RE3. Indeed, the seminiferous tubule area significantly decreased from D0 to D7 with RE3 (p=0.003). RE3 appeared to be deleterious for seminiferous tubule growth as well as RE4 and RERA5; these conditions impaired significantly the seminiferous tubule growth after 11 days of organotypic culture compared with the other conditions tested (p<0.01). After exclusion of the above mentioned conditions, seminiferous tubules area was significantly higher after 7 days of culture, for RE6 compared to RARE6 and RARE5 (p=0.02 for both) but was similar between RE6 and RERA6. After 9 days of culture, a similar seminiferous tubule area was observed with RE6, RERA6, RARE6 and RARE5; indeed, the greatest seminiferous tubule area was obtained on D11 with these culture conditions without significant differences (5907.2±545.90µm^2^, 5641.7±439.19µm^2^, 5478.3±943.39µm^2^ and 5094.4±332.00µm^2^ respectively).

**Figure 1:**
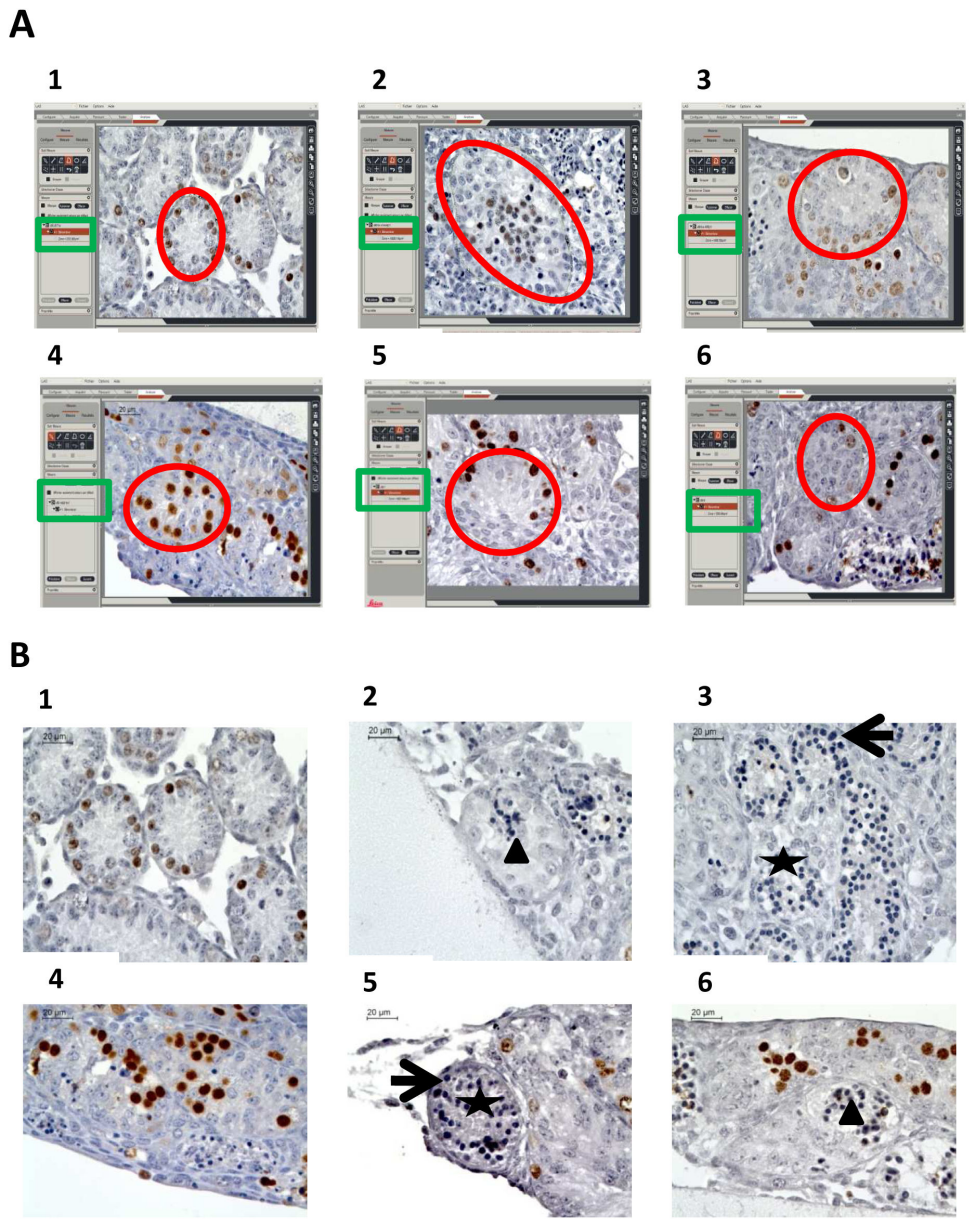
Seminiferous tubule growth (A) and intra-tubular necrosis (B) during organotypic culture of prepubertal mice testes under the different culture conditions assessed. (A) Seminiferous tubule area was defined using software (red circle) and area was automatically generated (green rectangle). (B) The percentage of partially (arrow head) or totally (star) necrotic seminiferous tubules were assessed as well as number of pyknotic nuclei (head). **(1-2-3)** Organotypic culture of fresh immature mice testicular tissue after 0 (1) or 11 (2 and 3) days of culture with RE6 (2) or RERA5 (3) **(4-5-6)** Organotypic culture of frozen-thawed testicular tissue with stabilisation phase at –8°C (5) and -9°C (6) compared to fresh control organotypic culture (4) Footnotes: **RE6**: 10^-6^M retinol ; RERA5: 10^-5^M retinoic acid and 3.3.10^-7^M retinol

**Figure 2:**
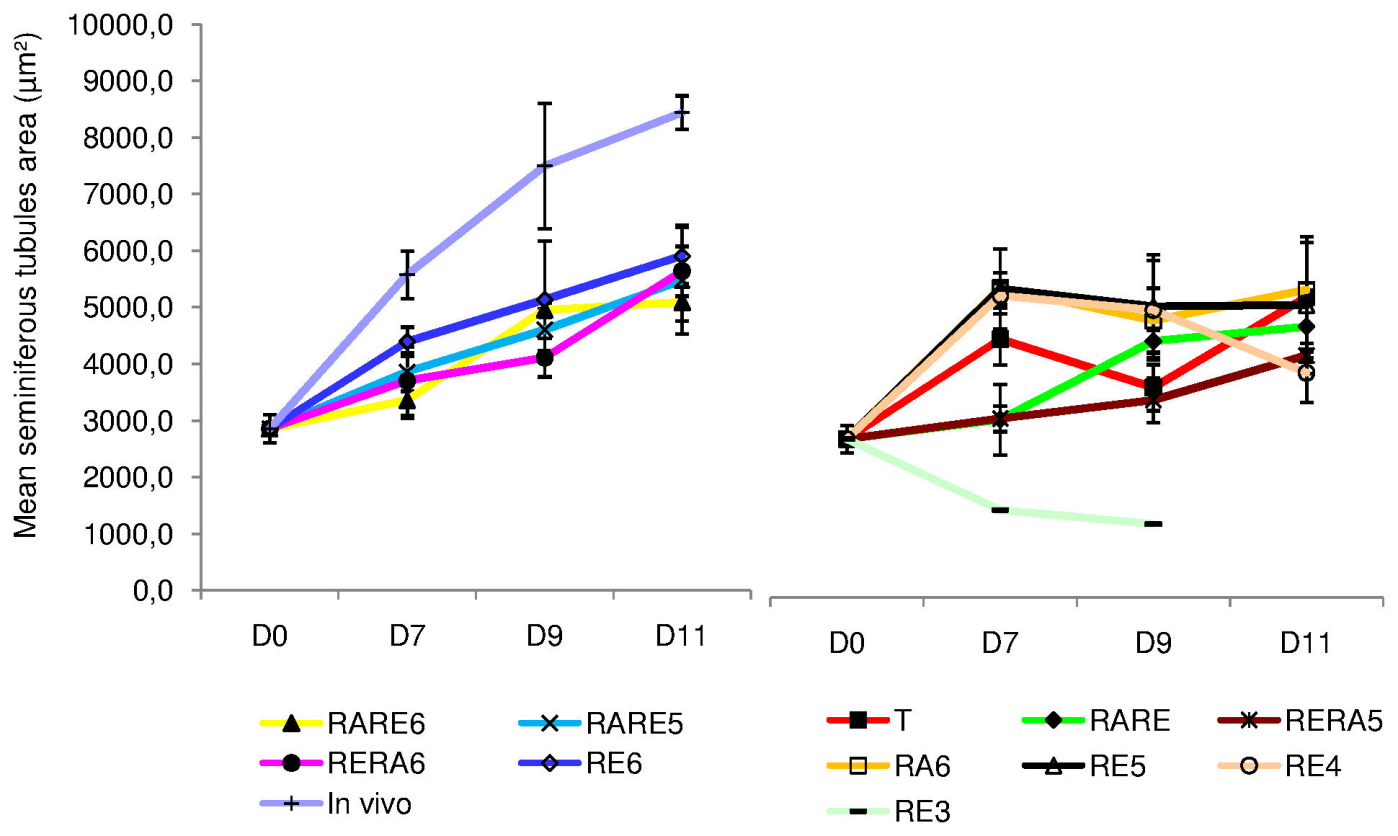
Seminiferous tubule area assessment in organotypic culture of fresh pre-pubertal mouse testicular tissue. Seminiferous tubule area evolution after 0, 7, 9 and 11 days of organotypic culture on at least 30 cross-sectioned tubules after Tra98 immunostaining under the different culture conditions. The results are represented as the mean ± SEM with n=6. Footnotes: T: basal culture medium without retinoid; **RARE**: 3.3.10^-7^M RA and 3.3.10^-7^M RE; **RARE6**: 3.3.10^-7^M RA and 10^-6^M RE; **RARE5**: 3.3.10^-7^M RA and 10^-5^M RE; **RERA6**: 3.3.10^-7^M RE and 10^-6^M RA; **RERA5**: 3.3.10^-7^M RE and 10^-5^M RA; **RA6**: 10^-6^M RA; **RE6**: 10^-6^M RE; **RE5**: 10^-5^M RE; **RE4**: 10^-4^M RE; **RE3**: 10^-3^M RE; **In** **vivo**: In vivo control.

**RA**: Retinoic acid; **RE**: Retinol; **D0**: Day 0; **D7**: Day 7; **D9**: Day 9; **D11**: Day 11

#### Cellular death (Figure 1B_1_ to 1B_3_ and Figure 3)

Partial or total necrotic seminiferous tubule proportions increased significantly between D0 and D11 regardless of the culture medium used (p<0.05). This phenomenon was mostly observed in the periphery of the testicular pieces. For RE3, all the seminiferous tubules were necrotic after 7 days of culture; thus, we excluded this condition for further analyses. RERA5, RE5, RA6, RE6 and T showed the higher proportions of total necrotic seminiferous tubules after 11 days of culture (44.5±1.27%, 33.7±0.33%, 34.0±0.00%, 30.0±8.00%, 29.0±9.00% respectively). Furthermore, among these five conditions, RERA5 appeared to be the more deleterious condition (p<0.01). Whatever the time point in culture, RE4, RARE, RARE6, RARE5 and RERA6 reduced seminiferous tubule necrosis compared with T, RERA5, RE5, RE6 and RE3 without significant difference, (6.0±6.00%, 7.2±1.79%, 15.0±11.77%, 16.6±1.96%, 18.3±11.08% respectively after 11 days of culture). Seminiferous tubules with partial necrosis were also more frequently observed with RERA5, RE5, RA6, RE6, T as well as RE4 conditions (data not shown).

**Figure 3:**
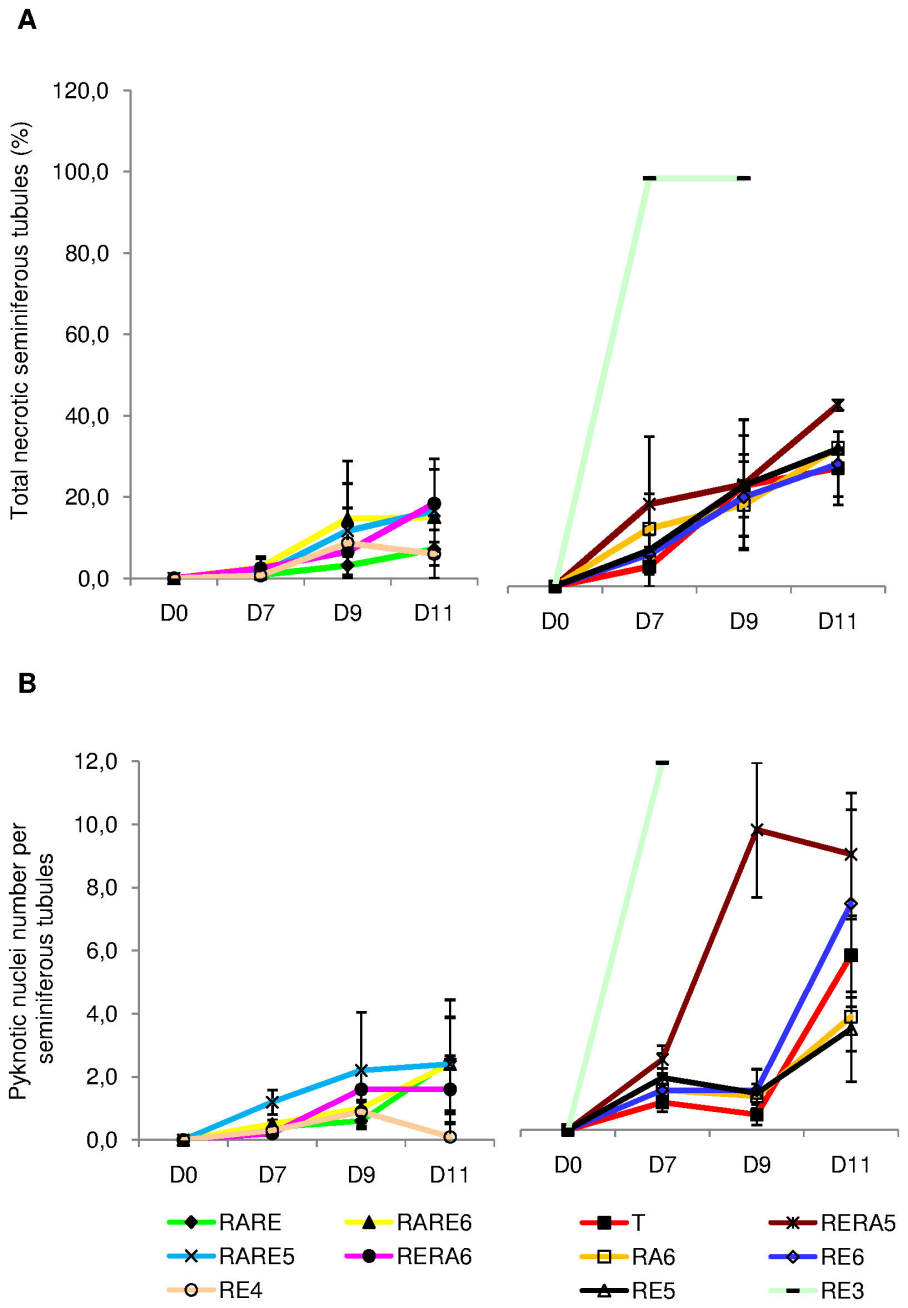
Cellular death assessment in organotypic culture of fresh pre-pubertal mouse testicular tissue. Cellular death was assessed in organotypic culture at days 0, 7, 9 and 11 on 30 cross-sectioned seminiferous tubules after Tra98 immunolabelling under the different culture conditions tested. (A) Percentage of total pyknotic seminiferous tubules. (B) Number of pyknotic nuclei per seminiferous tubules. The results are represented as the mean ± SEM with n=6. Footnotes: T: basal culture medium without retinoid; **RARE**: 3.3.10^-7^M RA and 3.3.10^-7^M RE; **RARE6**: 3.3.10^-7^M RA and 10^-6^M RE; **RARE5**: 3.3.10^-7^M RA and 10^-5^M RE; **RERA6**: 3.3.10^-7^M RE and 10^-6^M RA; **RERA5**: 3.3.10^-7^M RE and 10^-5^M RA; **RA6**: 10^-6^M RA; **RE6**: 10^-6^M RE; **RE5**: 10^-5^M RE; **RE4**: 10^-4^M RE; **In** **vivo**: In vivo control. **RA**: Retinoic acid; **RE**: Retinol; **D0**: Day 0; **D7**: Day 7; **D9**: Day 9; **D11**: Day 11

As a consequence, the number of pyknotic nuclei increased significantly between 0 and 11 days of culture (Figure 3B). Indeed, RERA5, RE6, T, RA6 and RE5 appeared to be the most deleterious conditions whereas RE4, RERA6, RARE5, RARE6 and RARE seemed to have less pyknotic nuclei. However, despite no significant difference, the number of pyknotic nuclei was higher for RERA5 between 7 and 9 days of culture, while for all other conditions, pyknotic nuclei were more numerous between D9 and D11. After 11 days of culture, RERA5 presented significantly more pyknotic nuclei than the other four deleterious conditions (p<0.01) while RE4 presented significantly less pyknotic nuclei than the other conditions (p=0.01). Whatever the culture conditions, the distinction between necrotic Sertoli cells and necrotic germ cells was not possible because necrotic cells were not immunostained with Tra98 monoclonal antibody (Figure S1). 

#### Intra-tubular cell proliferation (Figure 4A, 5 and 6)

PCNA expression decreased significantly between D0 and D7 under all the retinoid concentrations tested (p=0.03), then tended to stabilization from D7 to D11 for T, RA6 and RERA5 or continued to decrease significantly for the other conditions (p<0.05) (Figure 5A). The significant decrease observed between D0 and D7 corresponded to Sertoli cell proliferation arrest as shown in Figure 5B. Indeed, Sertoli cell proliferation significantly decreased for all the culture conditions tested (p=0.01) whereas germ cell proliferation remained globally stable between D0 and D7 (Figure 5B and 5C).

**Figure 4:**
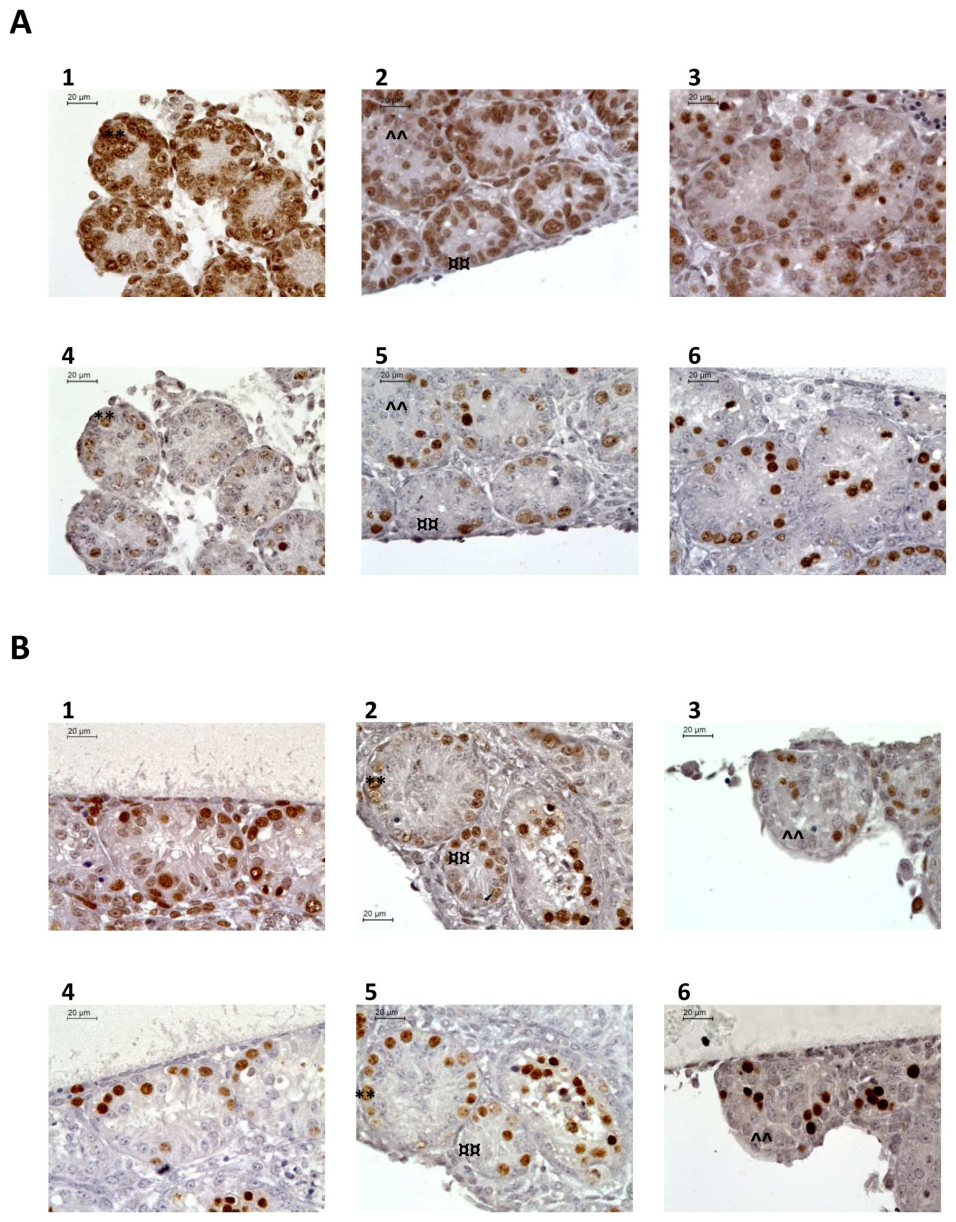
Assessment of cellular proliferation after PCNA (Proliferating Cell Nuclear Antigen) and Tra98 immunodetection on serial sections of seminiferous tubules in organotypic culture of fresh and frozen-thawed pre-pubertal mouse testicular tissue. (A) Cellular proliferation assessment for organotypic culture of fresh immature mice testicular tissue assessed after 0 (1 and 4) or 11 days (2,3,5,6) of culture with RE6 (2 and 5) or RERA5 (3 and 6) (B) Cellular proliferation assessment for organotypic culture of frozen-thawed testicular tissue with stabilisation phase at –8°C (2 and 5) and -9°C (3 and 6) compared to fresh control organotypic culture (1 and 4) **1** **to 3** : PCNA immunodetection **4** **to 6** : Tra98 iommunostaining ** : Germ cells PCNA positive ; **¤¤** : Sertoli cell PCNA positive ; **^^** : Sertoli cell PCNA negative Footnotes: **RE6** : 10^-6^M retinol ; **RERA5** : 10^-5^M retinoic acid and 3.3.10^-7^M retinol

**Figure 5:**
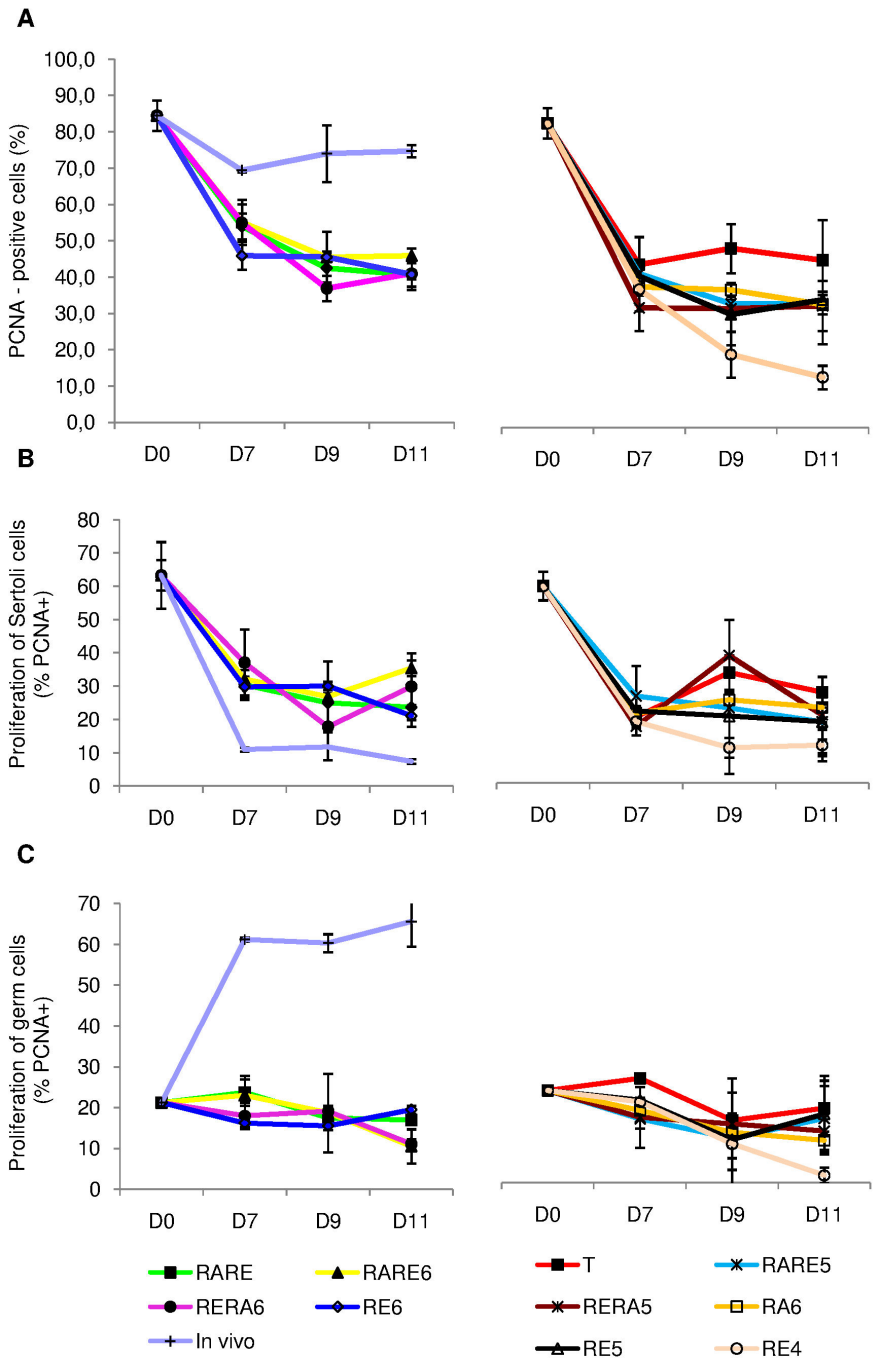
PCNA (Proliferating Cell Nuclear Antigen) expression in organotypic culture of fresh pre-pubertal mouse testicular tissue. PCNA expression was assessed in organotypic culture at days 0, 7, 9 and 11 on at least 500 intra-tubular cells under the different culture conditions tested. The results are represented as the mean ± SEM with n=6. (A) Percentage of total PCNA-positive cells. (B) Percentage of PCNA-positive Sertoli cells. (C) Percentage of PCNA-positive germ cells. FootnotesT: basal culture medium without retinoid; **RARE**: 3.3.10^-7^M RA and 3.3.10^-7^M RE; **RARE6**: 3.3.10^-7^M RA and 10^-6^M RE; **RARE5**: 3.3.10^-7^M RA and 10^-5^M RE; **RERA6**: 3.3.10^-7^M RE and 10^-6^M RA; **RERA5**: 3.3.10^-7^M RE and 10^-5^M RA; **RA6**: 10^-6^M RA; **RE6**: 10^-6^M RE; **RE5**: 10^-5^M RE; **RE4**: 10^-4^M RE; **In** **vivo**: In vivo control. **RA**: Retinoic acid; **RE**: Retinol; **D0**: Day 0; **D7**: Day 7; **D9**: Day 9; **D11**: Day 11

**Figure 6:**
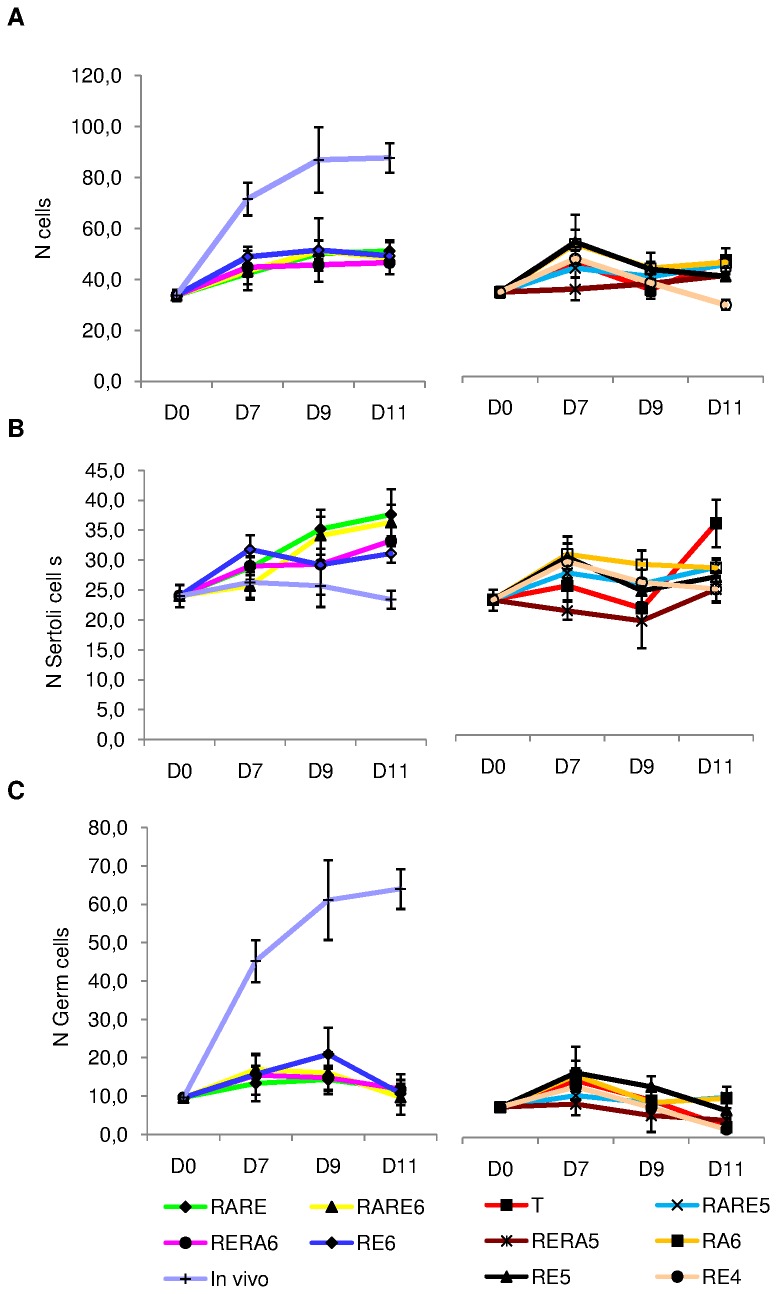
Cellular proliferation in organotypic culture of fresh pre-pubertal mouse testicular tissue on days 0, 7, 9 and 11. Intratubular cell quantification (germ cells and Sertoli cells) was assessed after Tra98 immunodetection on 30 cross-sectioned seminiferous tubules for each tested concentration of retinoids. The results are represented as the mean ± SEM with n=6. (A) Total number of intratubular cells. (B) Number of Sertoli cells per seminiferous tubules. (C) Number of germ cells per seminiferous tubules. Footnotes: T: basal culture medium without retinoid; **RARE**: 3.3.10^-7^M RA and 3.3.10^-7^M RE; **RARE6**: 3.3.10^-7^M RA and 10^-6^M RE; **RARE5**: 3.3.10^-7^M RA and 10^-5^M RE; **RERA6**: 3.3.10^-7^M RE and 10^-6^M RA; **RERA5**: 3.3.10^-7^M RE and 10^-5^M RA; **RA6**: 10^-6^M RA; **RE6**: 10^-6^M RE; **RE5**: 10^-5^M RE; **RE4**: 10^-4^M RE; **In** **vivo**: In vivo control. **RA**: Retinoic acid; **RE**: Retinol; **D0**: Day 0; **D7**: Day 7; **D9**: Day 9; **D11**: Day 11

After 7 days of culture, PCNA expression was significantly higher for RERA6 compared to RE6, T, RERA5, RA6, RE5 and RE4. However, at D9, PCNA expression was significantly higher for RE6 compared to RERA6, RERA5, RA6, RE5 and RE4 (p=0.004). RE4 appeared to be deleterious for cellular proliferation in pre-pubertal mouse testicular tissue culture whereas the nine other conditions gave a similar PCNA expression after 11 days of culture (p>0.05) (Figure 5A).

The intra-tubular cell number increased significantly between D0 and D7 for T, RA6, RE6, RE4 and RARE6 (p=0.03) but did not vary for RE5, RARE, RARE5, RERA6, RERA5 (Figure 6A). This increase stopped at D9 and then stabilized until D11 for RE6 and RARE6. For RARE and RERA6, intra-tubular cell number was not modified between D7 and D11. No significant difference was observed between RE6, RARE6, RARE and RERA6 conditions after 11 days of culture (51.1±4.28, 49.2±1.60, 48.4±6.27, 46.6±1.15). On the other hand, RE5, RA6, T and RE4 gave a significant decrease of intra-tubular cell number between D7 and D9 (p=0.03). RERA5 induced no change between D0 and D11. Whatever the time point of culture, no significant difference was observed between the different culture conditions when compared with each other.

The ratio between Sertoli cells and spermatogonia increased significantly between D0 and D11 for all the culture conditions tested (p=0.0105) while this ratio decreased significantly in seminiferous tubules of mice aged between 7 days post-partum and 18 days post-partum (p=0.0039). Furthermore, the ratio was significantly higher at D11 in vitro compared to the in vivo controls whatever the culture conditions tested (p=0.0105). The ratio increased significantly between D0 and D7 for RE6 (p=0.0039) but did not vary for the other culture conditions. On the other hand, the ratio did not vary between D7 and D11 for RE6 but increased significantly for the other culture conditions (p=0.0105) except for RARE5. At D11, the ratio was not different between RE6 and each other culture condition (Figure S2).

#### Assessment of undifferentiated (Plzf+) and differentiated (c-kit+) spermatogonia

Plzf and c-kit were not always and not simultaneously expressed in vivo during the post-natal development of the testis. However, Plzf was expressed in spermatogonia at 7, 14, 16 and 18 dpp and c-kit at 7 dpp and 9 dpp respectively. Undifferentiated spermatogonia (plzf+) were detected in vitro in one assay only at D7 for AR5 (5.83%) and AR6 (0.52%) respectively, at D9 for RE5 (60.1%) and at D11 for basal medium (6.22%). Furthermore, differentiated spermatogonia (c-kit+) were observed in one assay only at D9 (60.1%) and D11 (3.83%) for RE5 respectively (Figures S3 and S4).

#### Assessment of meiotic germ cells (Figure *7* and *8*)

The assessment of meiotic germ cell was performed using (i) the percentage of seminiferous tubules at meiotic prophase i.e. containing spermatocytes I (leptotene, zygotene or pachytene) (Figure 8A) and (ii) the percentage of meiotic cells in these seminiferous cords (Figure 8B). The initiation of spermatogenesis was characterized by the presence of germ cells at prophase I of meiosis and was observed for all the culture conditions tested. Indeed, the percentage of seminiferous tubules at prophase I of meiosis increased significantly between D0 and D7 for each condition tested (p=0.03), continued to progress until D9 for RARE, RE6, RERA6, RARE6, RARE5 and RERA5, although this increase was lower for RERA5. On the other hand, the percentage of seminiferous tubules at meiosis I decreased significantly from D7 to D9 for RA6 and RE4 (p=0.03 for both). Thereafter, this percentage tended to stabilisation. The percentage of seminiferous tubules at prophase I of meiosis was higher at D11 for RE6, RARE, RERA6 and RARE5 without significant difference (66.9±2.36%, 70.5±7.17%, 67.0±5.48%, 66.8±16.48% respectively, p>0.05) compared to the other conditions. However, after D7, the percentage of seminiferous tubules at meiosis I was significantly higher for RE6 compared to RARE and RARE5 (p=0.02 and p=0.004 respectively) but was similar at D9 for RE6, RARE, RERA6 and RARE5 (Figure 8A).

**Figure 7:**
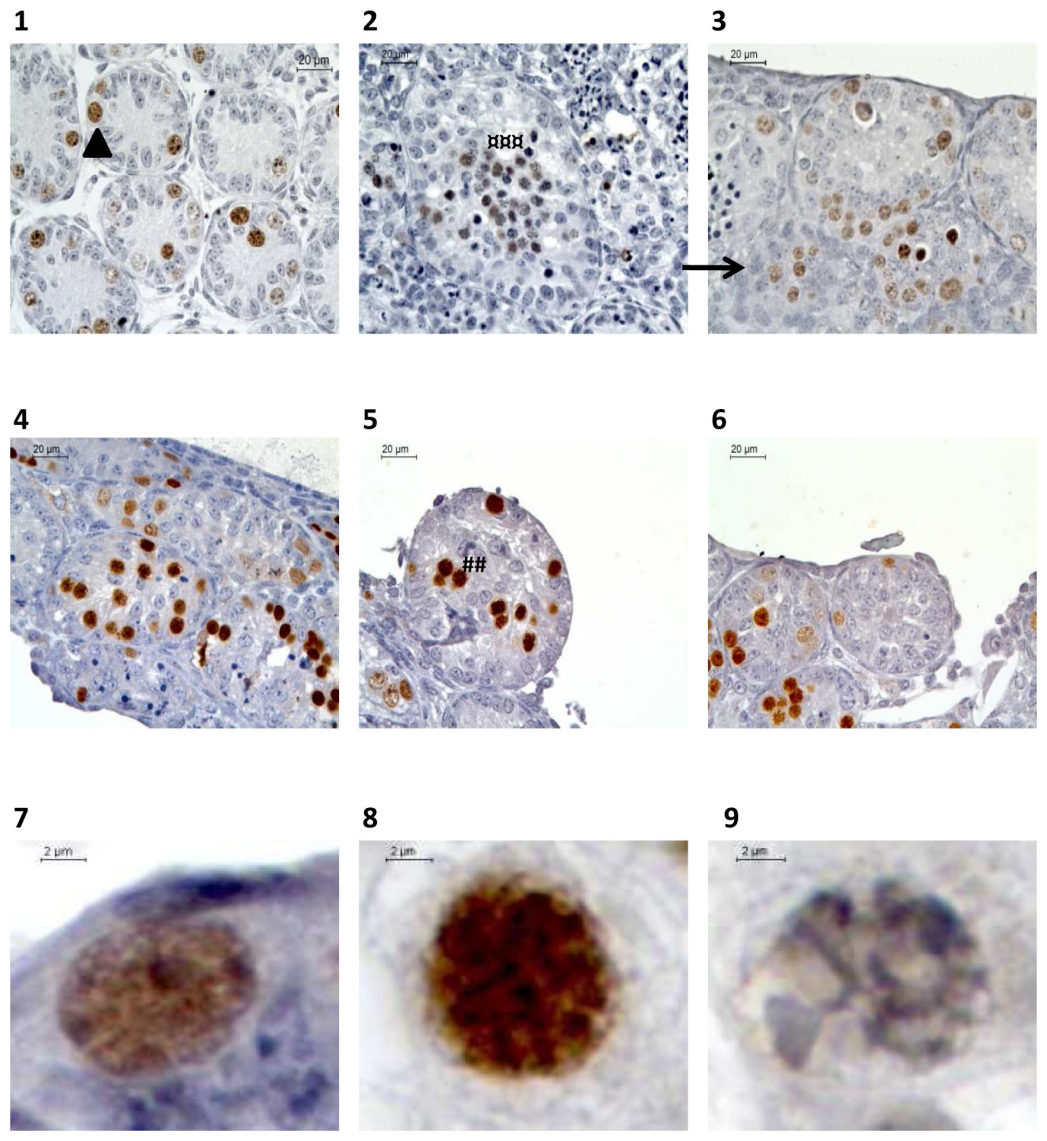
Histological evaluation of meiotic process evaluation after Tra98 immunostaining of organotypic culture of fresh and frozen-thawed pre-pubertal mice testicular tissue. (1-2-3) Meiotic germ cells assessment for organotypic culture of fresh pre-pubertal mice testicular tissue at ×500 magnification. Fresh testicular tissue was culture for 0 (1) or 11 days (2 and 3) with RE6 (2) or RERA5 (3) (4-5-6) Meiotic germ cells assessment for organotypic culture of frozen-thawed (5 and 6) immature mice testicular tissue compared to fresh control (4) at ×500 magnification. Testicular tissue was frozen using a temperature stabilisation phase at –8°C (5) or –9°C (6) (7-8-9) Germ cells classification after Tra98 immunodtection at x1000 magnification. Intra-tubular cells were classified as spermatogonia (7, arrow head) (smooth spherical brown nuclei), leptotene/zygotene primary spermatocytes (8, ###) (irregular spherical brown nuclei with condensed chromatin) or pachytene primary spermatocytes (9, ¤¤¤) (irregular spherical brown nuclei with highly condensed chromatin). Sertoli cells was defined with a blue nucleus (head). Footnotes: RE6: 10^-6^M retinol ; RERA5: 10^-5^M retinoic acid and 3.3.10^-7^M retinol

**Figure 8:**
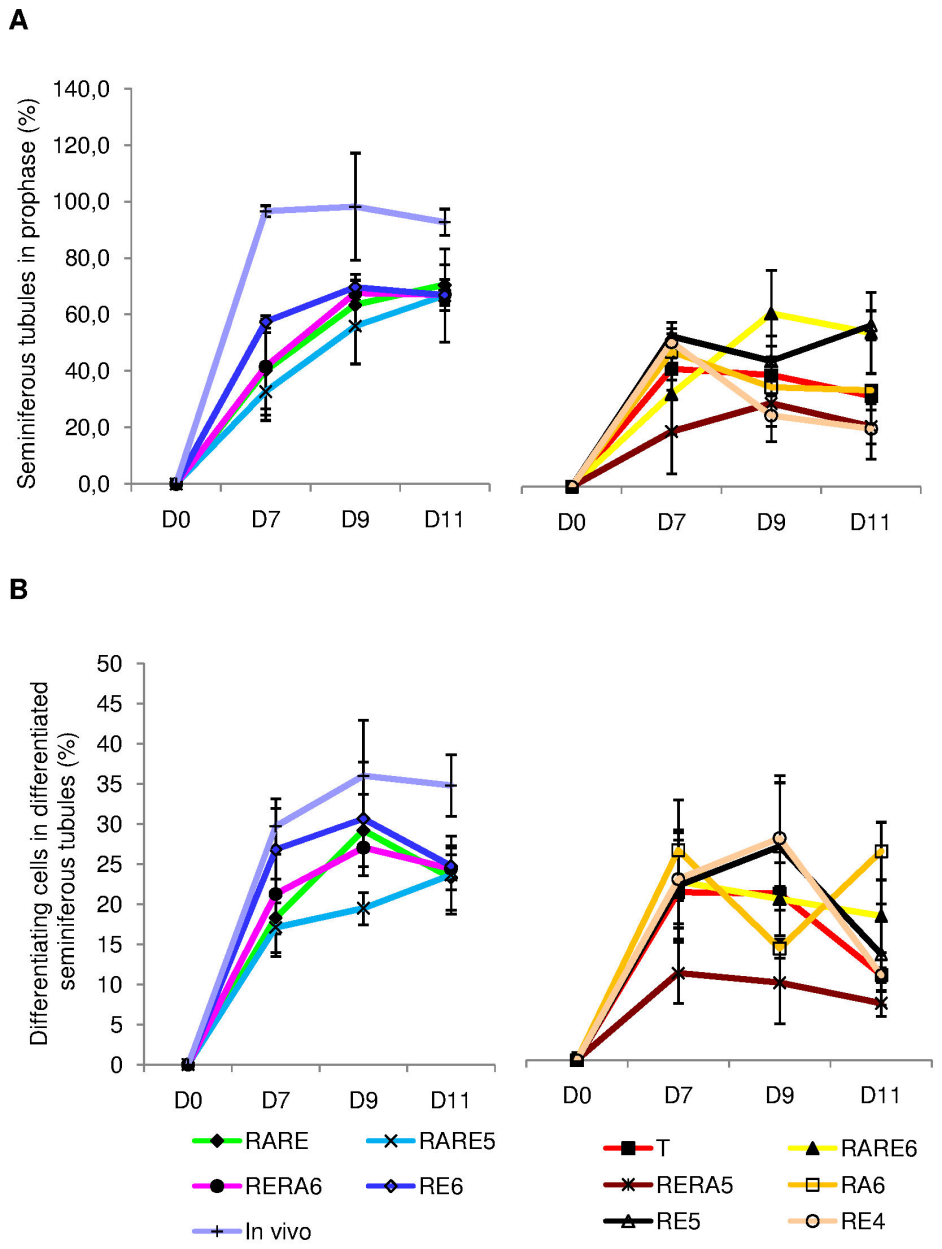
Meiotic process in organotypic culture of fresh pre-pubertal mouse testicular tissue at days 0, 7, 9 and 11. Meiotic germ cells assessment performed after Tra98 immunodetection on 30 cross-sectioned seminiferous for each tested concentration of retinoids. The results are represented as the mean ± SEM with n=6. (A) Percentage of seminiferous tubules in meiotic prophase (i.e containing leptotene, zygotene or pachytene primary spermatocytes). (B) Percentage of primary spermatocytes (i.e. leptotene, zygotene or pachytene) in meiotic seminiferous tubules. FootnotesT: basal culture medium without retinoid; **RARE**: 3.3.10^-7^M RA and 3.3.10^-7^M RE; **RARE6**: 3.3.10^-7^M RA and 10^-6^M RE; **RARE5**: 3.3.10^-7^M RA and 10^-5^M RE; **RERA6**: 3.3.10^-7^M RE and 10^-6^M RA; **RERA5**: 3.3.10^-7^M RE and 10^-5^M RA; **RA6**: 10^-6^M RA; **RE6**: 10^-6^M RE; **RE5**: 10^-5^M RE; **RE4**: 10^-4^M RE; **In** **vivo**: In vivo control. **RA**: Retinoic acid; **RE**: Retinol; **D0**: Day 0; **D7**: Day 7; **D9**: Day 9; **D11**: Day 11

In the seminiferous tubules with germ cells at prophase I of meiotic division, the mean percentage of meiotic cells increased between D0 and D9 for each medium tested (p<0.01) and then stabilized for RE6, RARE, RARE6, RARE5, RERA6, RERA5. As observed for the percentage of seminiferous tubules at meiotic prophase I, RE6, RARE, RERA6 and RARE5 allowed to produce a high rate of spermatocytes at prophase I without significant difference (24.8±1.45%, 23.3±4.02%, 24.4±2.62%, 23.6±4.87% respectively) (Figure 8B).

#### In Vitro Steroidogenesis (Figure 9)

Leydig cell functional integrity was well-preserved during fresh pre-pubertal mouse testicular tissue culture. Testosterone production increased significantly from D0 to D11 for each retinoid concentrations tested (p=0.03). RERA5, RE5 and RA6 gave the highest testosterone concentrations whereas RE4, RARE, RARE6 and RARE5 gave the lowest testosterone concentrations after D11. After D3 and D7, testosterone production was significantly lower for RERA5 compared to RE5 and RA6 (p=0.002 and p=0.003 respectively for D3; p=0.004 and p=0.02 respectively for D7) but was similar at D9. For the culture conditions with the lowest testosterone production, in vitro production of testosterone was significantly higher for RE4 compared to RARE, RARE6 and RARE5 at D3 (p=0.002 for the three different conditions), at D9 (p=0.004 for RARE and RARE5 and p=0.02 for RARE6) and at D11 (p=0.02 for RARE and RARE5 and p=0.004 for RARE6).

**Figure 9:**
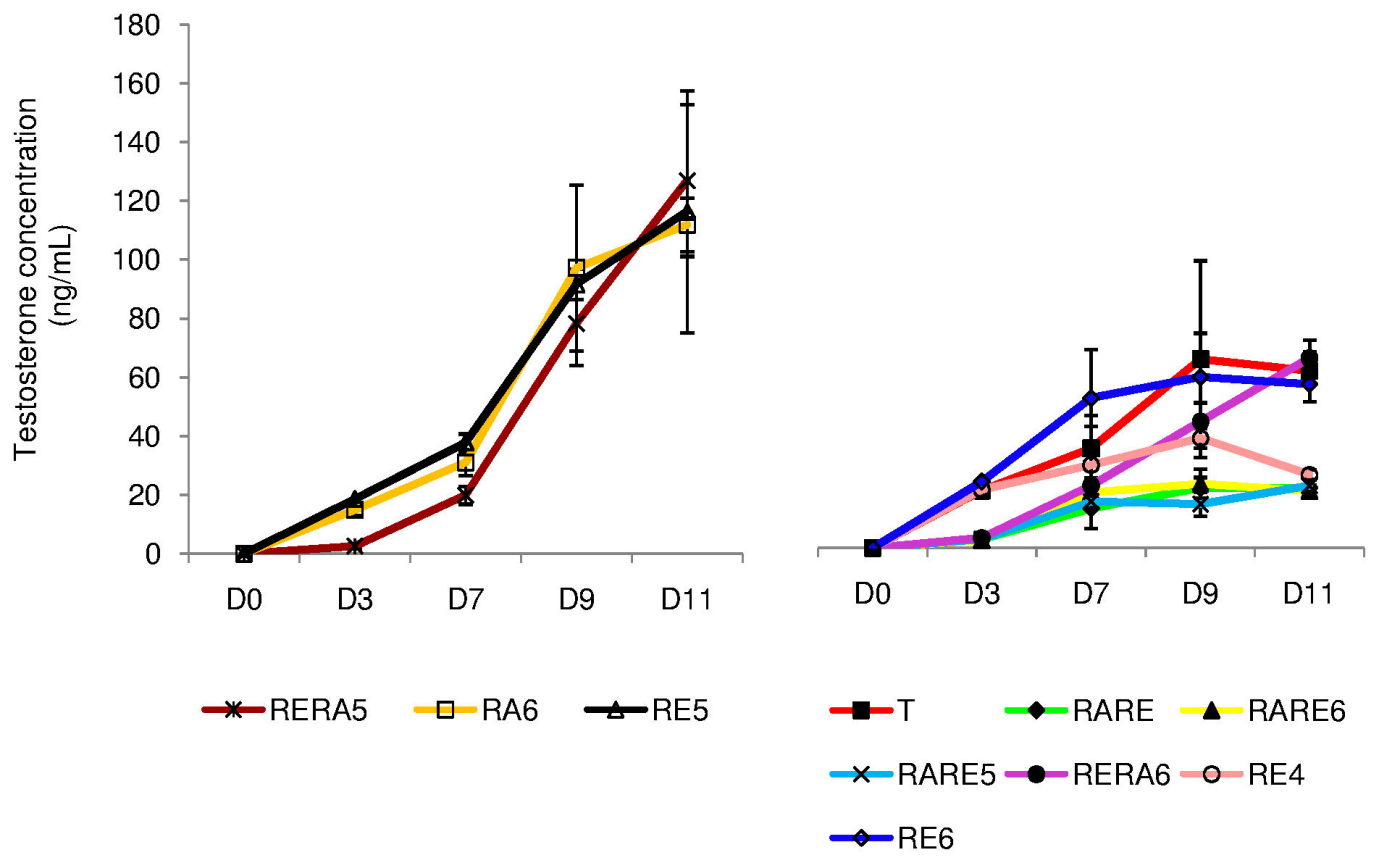
Evolution of testosterone concentrations (ng/mL) in culture media collected between D0 and D11. The results are represented as the mean ± SEM with n=6. Footnotes: T: basal culture medium without retinoid; **RARE**: 3.3.10^-7^M RA and 3.3.10^-7^M RE; **RARE6**: 3.3.10^-7^M RA and 10^-6^M RE; **RARE5**: 3.3.10^-7^M RA and 10^-5^M RE; **RERA6**: 3.3.10^-7^M RE and 10^-6^M RA; **RERA5**: 3.3.10^-7^M RE and 10^-5^M RA; **RA6**: 10^-6^M RA; **RE6**: 10^-6^M RE; **RE5**: 10^-5^M RE; **RE4**: 10^-4^M RE; **In** **vivo**: In vivo control. **RA**: Retinoic acid; **RE**: Retinol; **D0**: Day 0; **D7**: Day 7; **D9**: Day 9; **D11**: Day 11

### Synthesis of the data to highlight the best condition for organ culture of fresh pre-pubertal testicular tissue

As shown in Table 1, we highlighted the four best culture conditions after individual evaluation of the following parameters: the seminiferous tubule area, the ratio between Sertoli cells and spermatogonia, the cellular proliferation, the necrosis and the progression through the meiotic process. Only culture media supplemented with 10^-6^M Re or 10^-6^M RA and 3.3.10^-7^M Re were considered to be favourable for the normal growth of seminiferous tubules, the maintenance of intra-tubular cell proliferation and the progression of germ cells through the meiotic process. In addition, it was found that under the RE6 condition, each parameter evaluated separately followed a similar curve when compared to the in vivo controls (14-, 16- and 18-day-old mice respectively) (Figures 2 to 6). However, the mean seminiferous tubule area, the intra-tubular cell proliferation and the percentage of seminiferous tubules at meiotic stage were significantly higher for in vivo controls compared to cultured tissue (p<0.01), while the percentage of meiotic cells was not different (p>0.05). Furthermore, the ratio between Sertoli cells and spermatogonia increased significantly between D0 and D7 (p=0.0105) but remains stable between D7 and D11 (Figure S2).

**Table 1:** Culture media for fresh immature mouse testicular tissue offering the best results for each studied parameter (seminiferous tubule area, intra-tubular cell proliferation, necrosis, cellular differentiation) after 11 days of organotypic culture.

**Studied parameter**	**Most favourable culture condition**
Seminiferous tubule area	**RE6**, **RERA6**, RARE5, RARE6
Intra-tubular cell proliferation	RARE6, **RE6**, RARE, **RERA6**
Necrosis	RARE, RARE6
Cellular differentiation	RARE, **RE6**, **RERA6**, RARE5
Ratio between Sertoli cells and spermatogonia	**RE6**, **RARE5**

Footnotes:

**RARE**: 3.3.10-^7^M RA and 3.3.10-^7^M RE; **RARE6**: 3.3.10-^7^M RA and 10-^6^M RE; **RARE5**: 3.3.10-^7^M RA and 10-^5^M RE; **RERA6**: 3.3.10-^7^M RE and 10-^6^M RA; **RE6**: 10-^6^M RE.

**RA**: Retinoic acid; **RE**: Retinol

### Organ Culture of Frozen-Thawed Pre-Pubertal Mice Testicular Tissue (Figure 10)

Frozen-thawed pre-pubertal mouse testicular tissue, cryopreserved using a controlled slow freezing protocol with a soaking temperature evaluated at -7°C, -8°C or -9°C, was cultured for 9 days using RE6 condition. Seminiferous tubule area, cellular proliferation, necrosis, progression through meiotic process, the ratio between Sertoli cells and spermatogonia and testosterone production were evaluated and compared to fresh pre-pubertal mouse testicular tissue cultured with the same condition.

**Figure 10:**
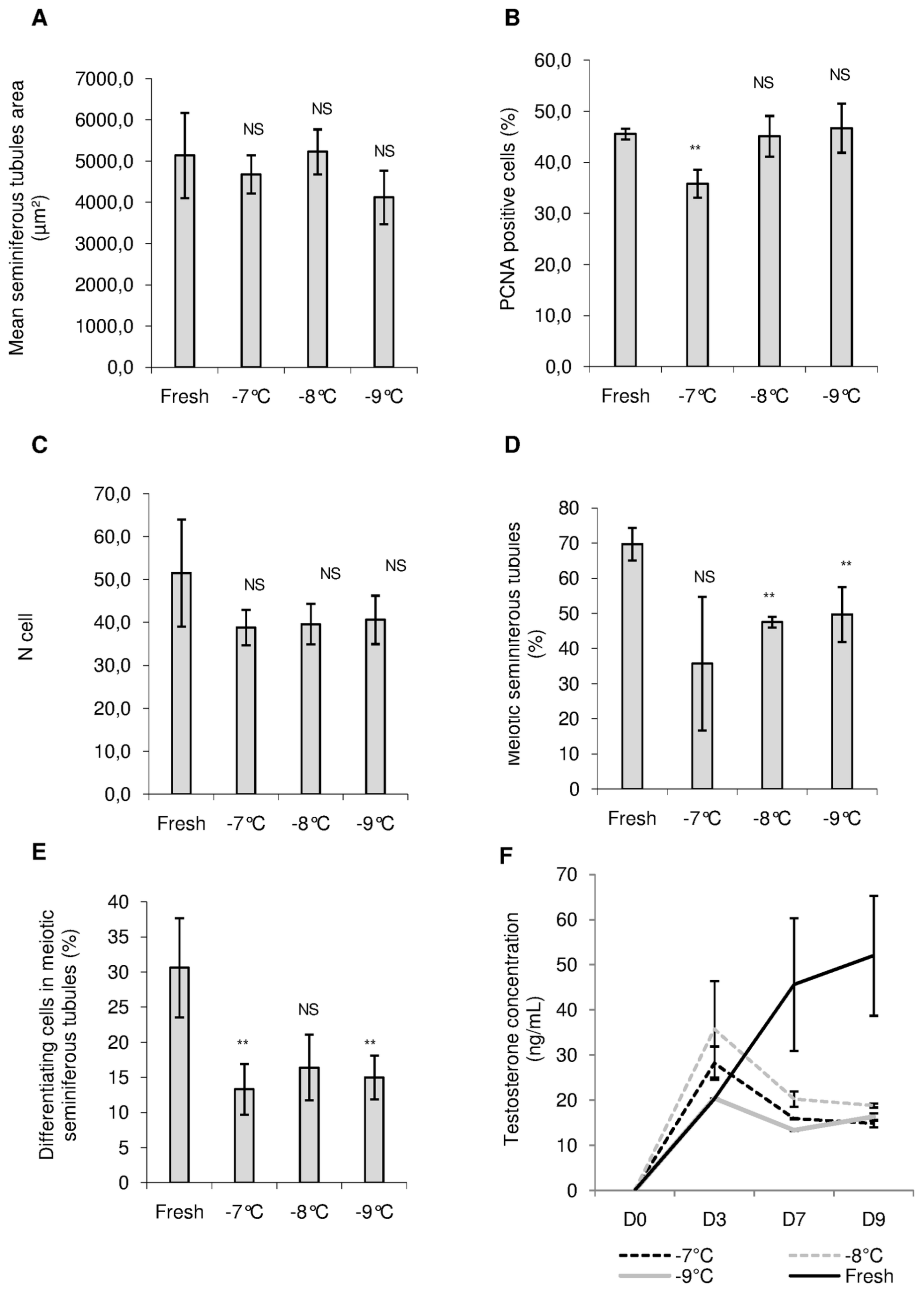
Structural and functional assessment of frozen-thawed pre-pubertal mouse testicular tissue after 9 days of culture with 10^-6^M retinol. Results were compared with fresh pre-pubertal testicular tissue cultured with the same conditions. Testicular tissue was cryopreserved using a controlled slow freezing protocol and a soaking temperature evaluated at -7°C, -8°C or -9°C. **(A)** Evolution of mean seminiferous tubule area on 30 cross-sectioned seminiferous tubules after Tra98 immunolabelling. **(B)** Cellular proliferation assessed by PCNA (Proliferating cell nuclear antigen) staining. **(C)** Quantification of intratubular cells after 9 days of culture using Tra98 immunodetection. **(D) and (E)** Meiosis assessment (i.e. leptotene, zygotene and pachytene spermatocytes I), after Tra98 immunostaining, using percentage of seminiferous tubules in meiotic prophase **(D)** or by assessment of percentage of primary spermatocytes (i.e. leptotene, zygotene or pachytene) in seminiferous tubules **(E)**. **(F)** Steroidogenesis evolution. The results are represented as the mean ± SEM with n=6. NS : Not significant ; ** : p<0.01

The mean seminiferous tubule area was similar for each soaking temperature tested (-7°C, -8°C and -9°C) compared to fresh control and compared to each other (p>0.05) (Figure 10A). PCNA expression was significantly lower for frozen-thawed cultured tissue cryopreserved with soaking temperature at -7°C compared to fresh cultured tissue (p<0.01). No statistical difference was observed for the two others soaking temperatures tested. Moreover, PCNA expression was significantly lower for thawed cultured tissue cryopreserved with soaking temperature at -7°C compared to the two others freezing conditions (p=0.02 and p=0.01 respectively) (Figure 10B). Furthermore, the intra-tubular cell number and the ratio between Sertoli cells and spermatogonia did not differ significantly between fresh and thawed cultured tissue and between each soaking temperature tested (Figure 10C and Figure S5 respectively).

After cryopreservation of pre-pubertal mouse testicular tissue, cells were able to undergo the meiotic process. Indeed, the percentage of seminiferous tubules at meiotic prophase (i.e. containing leptotene, zygotene or pachytene primary spermatocytes) was similar for a soaking temperature at -7°C compared to fresh control but was significantly reduced at -8°C and -9°C (p<0.01) (Figure 10D). No statistical difference was observed between each cryopreservation conditions. Although soaking temperature at -7°C appeared to be more favourable to obtain the best rate of seminiferous tubules in meiotic prophase, even if the standard error of mean was quite large, this condition was more deleterious in term of percentage of cells in meiotic prophase compared to fresh control. The same data were obtained for a soaking temperature at -9°C. A soaking temperature at -8°C gave a similar percentage of meiotic cells compared to fresh control (Figure 10E). However, no statistical difference was observed between each cryopreservation conditions. 

Whatever the freezing protocol tested, Leydig cells were able to synthesized testosterone as shown in Figure 10F. Androgen secretion assessed in culture media increased significantly, for each soaking temperature evaluated, between D0 and D9 (p=0.05). However, the mean testosterone concentration did not follow the same evolution as observed with control fresh tissue cultured under the same conditions. Steroidogenesis decreased between D3 and D7 then tended to stabilization for thawed mouse testicular tissue, whereas this parameter increased rapidly for control fresh tissue. A soaking temperature at -8°C gave a significant higher concentration of testosterone after 9 days of culture compared to -7°C and -9°C soaking temperature (p=0.02 for both).

## Discussion

This study demonstrated that retinol at a concentration of 10^-6^M provided the optimal culture conditions for in vitro maturation of fresh and frozen-thawed pre-pubertal spermatogonial stem cells.

Retinoid concentrations assessed in the current study were in agreement with reported concentrations of RE and RA measured in foetal and neonatal serum [31,32] and previously used for in vitro maturation of testicular tissue, foetal cells or embryonic cells [33-36]. A somatic and germ cell co-culture system, using seminiferous tubules from 23- to 25-day-old rats and a culture medium containing 3.3.10^-7^M RE and 3.3.10^-7^M RA, allowed the functional differentiation of pachytene spermatocytes from stages IV to VI into round spermatids over a 3-week period; however, the reproducibility of these experiments was not established [33]. Moreover, 10^-6^M RA triggered entry into meiosis of 11.5 and 12.5 dpc (days post-conception) XY mouse testis using organ culture system [35]. RA at a concentration of 10^-5^M was also used to develop a strategy for the establishment of spermatogonial stem cell lines from embryonic stem cells which were able to undergo meiosis and to generate haploid functional male gametes in vitro [34]. Furthermore, when RA was used at a concentration of 10^-7^M, foetal cattle male germ cells were able to differentiate into elongated spermatids [36].

Organ culture system, as used in our study, has already been assessed for testicular tissue in other animal models, for example in rat [17] and in humans [18,37], and has proven its efficiency for other types of tissue as adipose tissue [38] or heart [39]. This organotypic culture system maintains the polarity and the association of testicular cells; it also mimics normal seminiferous tubule compartmentalisation [14,40] and preserve the morphological interactions between intra-tubular cells and between intra-tubular cells and interstitial compartment that allow a better efficiency of the culture system than isolated cells or disintegrated testicular biopsies [37].

However, to our knowledge, no study has assessed the effect of RE or RA on in vitro proliferation and differentiation of spermatogonial stem cells from mouse pre-pubertal testis using organotypic culture system. In our fresh pre-pubertal mouse testicular tissue culture, only medium containing both 10^-6^M RA and 3.3.10^-7^M RE (RERA6) or 10^-6^M RE (RE6) appeared to be favourable for seminiferous tubule growth, maintenance of intra-tubular cell proliferation and progression through meiotic process. On the contrary, RE3, RE4, RERA5, RE5 and RA6 appeared to be deleterious for at least one of the different studied parameters. Retinoids played a major role during perinatal development in the regulation of cellular growth and differentiation. Indeed, in rat testis from 14.5- and 18.5-day-old foetuses and from 3-day-old neonates cultured for 3 days, 10^-6^M RA increased the mean diameter of the cords only for 18.5-dpc and 3-dpp testes whereas disrupt seminiferous cords were observed for 14.5-dpc cultured testes [21]. Furthermore, 10^-6^M RA also caused a decrease of the total number of gonocytes after 3 days of culture. In the present study, Plzf that triggers undifferentiated spermatogonia and c-kit that triggers differentiated spermatogonia were exceptionally expressed during the different culture conditions tested. This rare expression of spermatogonial markers was not due to a technical defect because both markers were expressed in the age-matched in vivo conditions [41,42]. Therefore, we were not able to evaluate specifically the impact of retinoids on spermatogonia self renewal and differentiation in our organ culture system. During spermatogenesis, Plzf and c-kit plays a key role in maintaining the balance between self renewal and differentiation of spermatogonial stem cells. Furthermore, Plzf directly acts by repressing the transcription of c-kit and c-kit is required in initiating the entry of spermatogonia into meiosis [42]. RA treatment reduced OCT4 and Plzf expression (markers of undifferentiated spermatogonia), caused stereotypical morphological changes and induced expression of c-kit, Stra8 (Stimulated by retinoic acid) and meiotic markers (SCP3, Rad51, γ-H2Aα) in mouse germ line stem cells [43,44] or in urogenital ridge organ cultures [45]. However, other studies showed that RA could not stimulate c-kit expression in spermatogenic cells and cause apoptosis of undifferentiated A_aligned_ spermatogonia [46]. We can propose that our organ culture system did not allow maintaining the pool of undifferentiated spermatogonia and that retinoids provoked enter into meiotic prophase of most differentiated spermatogonia in a synchronised manner. The decrease of the ratio between Sertoli cells and spermatogonia between D0 and D11 compared to the in vivo controls may also reinforce this hypothesis more specifically with 10^-6^ RE. Consequently, the continuous process of spermatogenesis was not maintained in agreement with the reduced number of intra-tubular germ cells observed after 9 days of culture whatever the culture conditions tested.

Vitamin A is critical for normal and continual sperm production, is clearly involved in type A spermatogonia proliferation, allows their differentiation into B spermatogonia and plays a major role for the entry into the meiotic process [47]. However, in our study, retinoid acted in a dose-dependent manner and retinol at a concentration of more than 10^-6^M stopped cellular proliferation and reduced quantitatively meiotic process. In embryonic stem cell culture, exogenous RA at a concentration of 10^-5^M allowed differentiation into primordial germ cells, then into gonocytes and lower RA concentrations (10^-6^M) were necessary to maintain spermatogenesis. However, to the best of our knowledge, these experiments were not reproduced [34].

Testosterone production was observed in the different fresh pre-pubertal testicular tissue culture conditions evaluated in our study, suggesting the maintenance of Leydig cell functional integrity. In rat and mouse foetal testis, retinoids have a negative impact on the onset of steroidogenesis whereas in adult rat testis, retinoids stimulate testosterone production by Leydig cells [19,21]. Moreover, testes of human foetuses under and after 7 week showed RA-stimulated and RA-independent testosterone secretion respectively [18]. However, high intratesticular levels of testosterone may interfere with spermatogonial proliferation and meiosis [48]. In our study, RA concentration of 10^-5^M in combination with 3.3.10^-7^M RE, RA at 10^-6^M or 10^-5^M RE gave to the highest testosterone level observed at D11, the highest rate of pyknotic nuclei, the highest rate of total necrotic seminiferous tubules and the lowest rates of meiotic cells. Leydig cell function impairment does not systematically induce intra-tubular cell alterations. This phenomenon was also observed when males were exposed to gonadoxic treatment that preferentially affected spermatogonial stem cells rather than Leydig cells. Testosterone production depends on the number and amount of seminiferous tubules and more precisely on the number amount of Leydig cells. In the current study, we used for each tested culture condition a 3mg whole prepubertal mice testis with a very probable and comparable number of seminiferous tubules between each culture condition. The impact of retinoids on endogenous testosterone synthesis observed in our organ culture system could be due to: (i) an increase of steroidogenesis enzyme gene expression in Leydig cells [18], this classical genomic pathway is generally considered as the main pathway used by androgens to exert their activity; (ii) an increase of enzymes involved in androgen synthesis which could occur by a non-genomic mechanism namely retinoylation (acylation of protein by retinoic acid), a high rate of retinoylation was shown on protein in Leydig cells line in the presence of 1.10^-7^M-2.10^-7^M RA [49].

Although pachytene stage has been achieved after retinoic stimulation in our organ culture system, we did not observe any differentiation up to post-meiotic germ cells which could have occurred with longer culture times. A reduced number of Sertoli cell or a defect of Sertoli cell maturation may affect the interaction with the germ cells and the normal establishment of blood testis barrier that becomes vital for germ cells survival and are essential for the normal progress of the differentiation pathway. Furthermore, defective retinoid signalling may induce, in part, an impairment of the blood testis barrier integrity. Indeed, several known RA-responsive gene encode structural components of tight and gap junctions [50].

The spermatogenic process impairment observed during the culture with increased necrotic intra-tubular cells could be explained by the fact that our organ culture system did not efficiently mimic the in vivo condition of spermatogenesis. Since pyknotic cells increased and intratubular germ cells decreased over time, all the spermatogonia might have progressively disappeared in our organ culture system. This hypothesis was proven for the different culture conditions tested except for RE6 and RARE5 that maintained the same ratio between Sertoli cells and spermatogonia between D7 and D11 of culture. The location of cellular death with more necrotic seminiferous tubules in the periphery of testicular piece may be explained by: (i) an inappropriate composition of the culture medium with a gradient of concentration of retinoids responsible for toxic effects on the periphery of the tissue and potentially sufficient and non toxic amount on the centre of the tissue, (ii) the culture system itself with a potential excess of medium which might partially immerse the tissue and reduce oxygenation. Indeed, pyknotic nuclei rates increased over time for each condition tested. Germ cell but also Sertoli cell damage could contribute to the decrease of seminiferous tubule growth and the impairment of germ cell differentiation.

In order to prevent this process and to improve in vitro spermatogenesis, our culture system should be improved using three-dimensional culture in agarose gel as proposed previously [49,50]. This type of culture allowed spermatids production using pup mouse testis tissues but further improvements are necessary considering that this progression up to spermatids was a rare event. We could also use a 3D coculture system in a collagen gel matrix which could enhance viability, meiosis and post-meiotic differentiation of germ cells [51-56]. These two culture systems allow a better assessment of medium level on testicular pieces. As a consequence, testicular tissue would be properly oxygenated and necrosis could be prevented.

In our fresh immature mouse testicular tissue cultures, medium containing both 10^-6^M RA and 3.3.10^-7^M RE or 10^-6^M RE were preferable for seminiferous tubule growth, maintenance of intra-tubular cell proliferation and germ cell differentiation and 10^-6^M RE was preferable for the stabilization of the ratio between Sertoli cells and spermatogonia after D7 of culture. In testicular tissue, RA is synthesized in situ and is predominantly produced from RE through a two-step metabolic pathway. RE is provided either by the blood where it circulates bound to the retinol-binding protein (RBP) or by the local storage of retinyl esters [57]. The first step in RE metabolism takes place in the peritubular cells, considered as the first RE cell targets. The peritubular cells take up the circulatory RE and secrete it as a complex formed with RBP in the direction of the Sertoli cells, main site of RA synthesis and also main site of RE storage (via RE esterification). RA synthesis from RE involves two steps catalyzed by alcohol dehydrogenases and retinaldehyde dehydrogenases and is then distributed to germ cells [20]. Aside from synthesis, degradation of RA is also a major balancing mechanism that protects cell from inadequate RA stimulation and is catalyzed by at least three cytochrome P450 hydroxylases (CYP26) [58]. However, it has been postulate that RE but not RA, which is degraded by CYP26 enzymes localized in peritubular myoid cells, passes freely from the blood circulation to the Sertoli cells and that blood-testis barrier inhibits the passage of RA circulating towards the germ cells [20,58]. In order to be close to physiological conditions in our culture system, we chose to use medium containing retinol at a concentration of 10^-6^M for our frozen-thawed pre-pubertal mouse testicular tissue culture. Furthermore, the different parameters explored in our study (seminiferous tubule growth, cell proliferation and meiosis) followed similar curves of evolution during the culture compared with the age-matched in vivo conditions.

Structural integrity of frozen-thawed pre-pubertal mouse testicular tissue seems to be well-preserved for a soaking temperature at -8°C and controlled slow freezing protocol without seeding as described previously [9,28]. Soaking temperature at -8°C allowed ensuring tissue functionality closed to fresh testicular tissue cultured with the same conditions. This temperature corresponds to water transition from liquid to solid phase and the withdrawal of water from the testicular tissue is absolutely necessary because the seminiferous tubules have the tendency to trap water that consequently induces ice formation during freezing. Ice formation would likely destroy the blood-testis barrier or tubule structure affecting post-freezing functionality and therefore, ice formation must be controlled and assessed [59]. Organotypic culture of frozen-thawed testicular tissue has shown to be a promising approach for in vitro maturation of pre-pubertal mouse [9,58] and pre-pubertal or young adult men [6,61,62] because it maintains testicular tissue architecture [6,9,60,61], spermatogonia survival [6,9,61] and Leydig cells ability to release testosterone [9,61]. However, except for published data by Milazzo et al. [9], in the different studies using freezing protocols included seeding at -7°C [60], -8°C [6,61] or -9°C [62], no retinoids were used in culture media during in vitro maturation of testicular tissue and for all the studies using organotypic culture of frozen-thawed testicular tissue, no spermatocytes I at meiotic prophase were observed. Furthermore, unlike the study designed by Milazzo et al. [9], our study assessed a pattern of retinoids concentration in order to improve in vitro maturation of fresh and frozen-thawed mouse testicular tissue. Moreover, we tried to improve freezing protocol by testing the effects of soaking temperature, a variable that was, to the best of our knowledge, not assessed in previous studies.

In conclusion, this study showed that RA and RE can control in vitro germ cell proliferation and differentiation. Retinol at a concentration of 10^-6^M is able to maintain germ cell proliferation and their ability to initiate spermatogenesis in fresh and frozen pre-pubertal mouse testicular tissue using a soaking temperature at -8°C. Moreover, Leydig cell functionality, as shown by LH-stimulated testosterone secretion in culture media, was maintained with retinol at a concentration of 10^-6^M, suggesting a possible human application for in vitro maturation of cryopreserved pre-pubertal testicular tissue.

## Supporting Information

Figure S1 **Histological evaluation using Tra98 immunostaining of seminiferous tubules obtained after organotypic culture of pre-pubertal mice testes at days 7 (**A**) and days 11 (**B**) of culture using a culture medium containing 10^-5^M retinoic acid.** Photomicrographs were captured at ×500 magnification.
Intra-tubular cells with pycnotic nuclei (black asterisks) were not stained with Tra98 antibody. The distinction between pycnotic Sertoli cells and pycnotic spermatogonia was not possible.
(TIF)

Figure S2 **Ratio between Sertoli cells and spermatogonia in organotypic culture of fresh pre-pubertal mouse testicular tissue.** The evolution of the ratio between Sertoli cells and spermatogonia was assessed after 0, 7, 9 and 11 days of organotypic culture on at least 30 cross-sectioned tubules after Tra98 immunostaining under the different culture conditions.
The results are represented as the mean ± SEM with n=6.
Footnotes:
T: basal culture medium without retinoid; **RARE**: 3.3.10^-7^M RA and 3.3.10^-7^M RE; **RARE6**: 3.3.10^-7^M RA and 10^-6^M RE; **RARE5**: 3.3.10^-7^M RA and 10^-5^M RE; **RERA6**: 3.3.10^-7^M RE and 10^-6^M RA; **RERA5**: 3.3.10^-7^M RE and 10^-5^M RA; **RA6**: 10^-6^M RA; **RE6**: 10^-6^M RE; **RE5**: 10^-5^M RE; **RE4**: 10^-4^M RE; **RE3**: 10^-3^M RE; **In vivo**: In vivo control.
**RA**: Retinoic acid; **RE**: Retinol; **D0**: Day 0; **D7**: Day 7; **D9**: Day 9; **D11**: Day 11
(TIF)

Figure S3 **Immunohistochemistry with an antibody to Promyelocytic leukemia zinc finger (Plzf) (A-B) and an antibody to c-kit (C-D) on testicular tissue sections from 14 days post-partum (dpp) old mice and from organotypic culture at days 9 of culture using a culture medium containing 10^-6^M retinol.** Photomicrographs were captured at ×500 magnification. Brown stained undifferentiated (black asterisks) and differentiated (black arrows) spermatogonia were observed in seminiferous tubules of 14 dpp old mice and were not detected after organotypic culture of testicular tissue of pre-pubertal mice testes.
(TIF)

Figure S4 **Assessment of Promyelocytic leukemia zinc finger (Plzf) and c-kit expression in spermatogonia of mice seminiferous tubules from 7 days post partum (dpp) to 18 dpp.** The results are presented as the mean±SEM with n=2.
(TIF)

Figure S5 **Ratio between Sertoli cells and spermatogonia of frozen-thawed pre-pubertal mouse testicular tissue after 9 days of culture with 10^-6^M retinol.** Results were compared with fresh pre-pubertal testicular tissue cultured with the same conditions. Testicular tissue was cryopreserved using a controlled slow freezing protocol and a soaking temperature evaluated at -7°C, -8°C or -9°C.
(TIF)

## References

[1] Dohle GR (2010) Male infertility in cancer patients: Review of the literature. Int J Urol 17: 327-331. doi:10.1111/j.1442-2042.2010.02484.x. PubMed: 20202000. 20202000

[2] Schrader M, Müller M, Straub B, Miller K (2001) The impact of chemotherapy on male fertility: a survey of the biologic basis and clinical aspects. Reprod Toxicol 15: 611-617. doi:10.1016/S0890-6238(01)00182-4. PubMed: 11738514. 11738514

[3] Tournaye H, Goossens E, Verheyen G, Frederickx V, De Block G et al. (2004) Preserving the reproductive potential of men and boys with cancer: current concepts and future prospects. Hum Reprod Update 10: 525-532. doi:10.1093/humupd/dmh038. PubMed: 15319377. 15319377

[4] Menon S, Rives N, Mousset-Siméon N, Sibert L, Vannier JP et al. (2009) Fertility preservation in adolescent males: experience over 22 years at Rouen University Hospital. Hum. Reprod 24: 37-44. 10.1093/humrep/den361 18945713

[5] Ginsberg JP, Carlson CA, Lin K, Hobbie WL, Wigo E et al. (2010) An experimental protocol for fertility preservation in prepubertal boys recently diagnosed with cancer: a report of acceptability and safety. Hum Reprod 25: 37-41. doi:10.1093/humrep/dep371. PubMed: 19861330. 19861330 PMC2794668

[6] Keros V, Hultenby K, Borgström B, Fridström M, Jahnukainen K et al. (2007) Methods of cryopreservation of testicular tissue with viable spermatogonia in pre-pubertal boys undergoing gonadotoxic cancer treatment. Hum Reprod 22: 1384-1395. doi:10.1093/humrep/del508. PubMed: 17259225. 17259225

[7] Wyns C, Curaba M, Petit S, Vanabelle B, Laurent P et al. (2011) Management of fertility preservation in prepubertal patients: 5 years' experience at the Catholic University of Louvain. Hum. Reprod 26: 737-747. 10.1093/humrep/deq387 21227939

[8] Ogawa T, Ohmura M, Ohbo K (2005) The niche for spermatogonial stem cells in the mammalian testis. Int J Hematol 82: 381-388. doi:10.1532/IJH97.05088. PubMed: 16533739. 16533739

[9] Milazzo JP, Vaudreuil L, Cauliez B, Gruel E, Massé L et al. (2008) Comparison of conditions for cryopreservation of testicular tissue from immature mice. Hum Reprod 23: 17-28. doi:10.1093/humrep/den1008. PubMed: 17989070. 17989070

[10] Jahnukainen K, Ehmcke J, Söder O, Schlatt S (2006) Clinical potential and putative risks of fertility preservation in children utilizing gonadal tissue or germline stem cells. Pediatr Res 59: 40R-47R. doi:10.1203/01.pdr.0000205153.18494.3b. PubMed: 16549547. 16549547

[11] Wyns C, Curaba M, Vanabelle B, Van Langendonckt A, Donnez J (2010) Options for fertility preservation in prepubertal boys. Hum Reprod Update 16: 312-328. doi:10.1093/humupd/dmp054. PubMed: 20047952. 20047952

[12] Hou M, Andersson M, Eksborg S, Söder O, Jahnukainen K (2007) Xenotransplantation of testicular tissue into nude mice can be used for detecting leukemic cell contamination. Hum Reprod 22: 1899-1906. doi:10.1093/humrep/dem085. PubMed: 17452397. 17452397

[13] Patience C, Takeuchi Y, Weiss RA (1998) Zoonosis in xenotransplantation. Curr Opin Immunol 10: 539-542. doi:10.1016/S0952-7915(98)80220-3. PubMed: 9794833. 9794833

[14] Parks JE, Lee DR, Huang S, Kaproth MT (2003) Prospects for spermatogenesis in vitro. Theriogenology 59: 73-86. doi:10.1016/S0093-691X(02)01275-X. PubMed: 12499019. 12499019

[15] Rouiller-Fabre V, Levacher C, Pairault C, Racine C, Moreau E et al. (2003) Development of the foetal and neonatal testis. Andrologia 35: 79-83. doi:10.1046/j.1439-0272.2003.00540.x. PubMed: 12558532. 12558532

[16] Staub C (2001) A century of research on mammalian male germ cell meiotic differentiation in vitro. J Androl 22: 911-926. PubMed: 11700854. 11700854 10.1002/j.1939-4640.2001.tb03430.x

[17] Livera G, Delbes G, Pairault C, Rouiller-Fabre V, Habert R (2006) Organotypic culture, a powerful model for studying rat and mouse fetal testis development. Cell Tissue Res 324: 507-521. doi:10.1007/s00441-006-0167-7. PubMed: 16520975. 16520975

[18] Lambrot R, Coffigny H, Pairault C, Donnadieu AC, Frydman R et al. (2006) Use of organ culture to study the human fetal testis development: effect of retinoic acid. J Clin Endocrinol Metab 91: 2696-2703. doi:10.1210/jc.2005-2113. PubMed: 16621909. 16621909

[19] Ghyselinck NB, Vernet N, Dennefeld C, Giese N, Nau H et al. (2006) Retinoids and spermatogenesis: lessons from mutant mice lacking the plasma retinol binding protein. Dev Dyn 235: 1608-1622. doi:10.1002/dvdy.20795. PubMed: 16586441. 16586441

[20] Livera G, Rouiller-Fabre V, Pairault C, Levacher C, Habert R (2002) Regulation and perturbation of testicular functions by vitamin A. Reproduction 124: 173-180. doi:10.1530/rep.0.1240173. PubMed: 12141930. 12141930

[21] Livera G, Rouiller-Fabre V, Durand P, Habert R (2000) Multiple effects of retinoids on the development of Sertoli, Germ, and Leydig cells of fetal and neonatal rat testis in culture. Biol Reprod 62: 1303-1314. doi:10.1095/biolreprod62.5.1303. PubMed: 10775181. 10775181

[22] Livera G, Pairault C, Lambrot R, Lelievre-Pegorier M, Saez JM et al. (2004) Retinoid-sensitive steps in steroidogenesis in fetal and neonatal rat testes: in vitro and in vivo studies. Biol Reprod 70: 1814-1821. doi:10.1095/biolreprod.103.021451. PubMed: 14960491. 14960491

[23] van Pelt AM, de Rooij DG (1990) Synchronization of the seminiferous epithelium after vitamin A replacement in vitamin A-deficient mice. Biol Reprod 43: 363-367. doi:10.1095/biolreprod43.3.363. PubMed: 2271719. 2271719

[24] Gaemers IC, van Pelt AM, van der Saag PT, de Rooij DG (1996) All-trans-4-oxo-retinoic acid: a potent inducer of in vivo proliferation of growth-arrested A spermatogonia in the vitamin A-deficient mouse testis. Endocrinology 137: 479-485. doi:10.1210/en.137.2.479. PubMed: 8593792. 8593792

[25] Morales C, Griswold D (1987) Retinol-induced synchronization in seminiferous tubules of the rat. Endocrinology 121: 432-434. doi:10.1210/endo-121-1-432. PubMed: 3595524. 3595524

[26] van Pelt AM, de Rooij DG (1991) Retinoic acid is able to reinitiate spermatogenesis in vitamin-A deficiënt rats and high replicate doses support the full development of spermatogenic cells. Endocrinology 128: 697-704. doi:10.1210/endo-128-2-697. PubMed: 1989855. 1989855

[27] Gaemers IC, Sonneveld E, van Pelt AM, Schrans BH, Themmen AP et al. (1998) The effect of 9-cis-retinoic acid on proliferation and differentiation of A spermatogonia and retinoid receptor gene expression in the vitamin-A deficient mouse testis. Endocrinology 139: 4269-4276. doi:10.1210/en.139.10.4269. PubMed: 9751509. 9751509

[28] Milazzo JP, Travers A, Bironneau A, Safsaf A, Gruel E et al. (2010) Rapid screening of cryopreservation protocols for murine prepubertal testicular tissue by histology and PCNA immunostaining. J Androl 31: 617-630. doi:10.2164/jandrol.109.009324. PubMed: 20203335. 20203335

[29] Travers A, Milazzo JP, Perdrix A, Metton C, Bironneau A et al. (2011) Assessment of freezing procedures for rat immature testicular tissue. Theriogenology 76: 981-990. doi:10.1016/j.theriogenology.2011.04.025. PubMed: 21664672. 21664672

[30] Tanaka H, Pereira LAVD, Nozaki M, Tsuchida J, Sawada K et al. (1997) A germ cell-specific nuclear antigen recognized by a monoclonal antibody raised against mouse testicular germ cells. Int J Androl 20: 361-366. PubMed: 9568529. 9568529 10.1046/j.1365-2605.1998.00080.x

[31] Berggren Söderlund MB, Fex GA, Nilsson-Ehle P (2005) Concentrations of retinoids in early pregnancy and in newborns and their mothers. Am J Clin Nutr 81: 633-636. PubMed: 15755833. 15755833 10.1093/ajcn/81.3.633

[32] Yang R, Li R, Mao S, Sun L, Huang X et al. (2007) The survey of serum retinol of the children aged 0-4 years in Zhejiang Province, China. BMC Public Health 7: 264-270. doi:10.1186/1471-2458-7-264. PubMed: 17892599. 17892599 PMC2071918

[33] Hue D, Staub C, Perrard-Sapori MH, Weiss M, Nicolle JC et al. (1998) Meiotic differentiation of germinal cells in three-week cultures of whole cell population from rat seminiferous tubules. Biol Reprod 59: 379-387. doi:10.1095/biolreprod59.2.379. PubMed: 9687311. 9687311

[34] Nayernia K, Lee JH, Drusenheimer N, Nolte J, Wulf G et al. (2006) Derivation of male germ cells from bone marrow stem cells. Lab Invest 86: 654-663. doi:10.1038/labinvest.3700429. PubMed: 16652109. 16652109

[35] Trautmann A, Guerquin MJ, Duquenne C, Lahaye JB, Habert R et al. (2008) Retinoic acid prevents germ cell mitotic arrest in mouse fetal testes. Cell Cycle 7: 656-664. doi:10.4161/cc.7.5.5482. PubMed: 18256537. 18256537

[36] Dong WZ, Hua JL, Shen WZ, Dou ZY (2010) In vitro production of haploid sperm cells from male germ cells of foetal cattle. Anim Reprod Sci 118: 103-109. doi:10.1016/j.anireprosci.2009.06.018. PubMed: 19632794. 19632794

[37] Roulet V, Denis H, Staub C, Le Tortorec A, Delaleu B et al. (2006) Human testis in organotypic culture: application for basic or clinical research. Hum Reprod 21: 1564-1575. doi:10.1093/humrep/del018. PubMed: 16497692. 16497692

[38] Toda S, Uchihashi K, Aoki S, Sonoda E, Yamasaki F, et al. (2009) Adipose tissue-organotypic culture system as a promising model for studying adipose tissue biology and regeneration. Organogenesis 5: 50-56. 19794899 10.4161/org.5.2.8347 PMC2710525

[39] Habeler W, Peschanski M, Monville C (2009) Organotypic heart slices for cell transplantation and physiological studies. Organogenesis 5: 62-66. 19794901 10.4161/org.5.2.9091 PMC2710527

[40] Livera G, Lambrot R, Frydman R, Coffigny H, Pairault C et al. (2007) Développement in vitro de la lignée germinale foetale male chez le rat, la souris et l’homme. Andrologie 17: 25-41. doi:10.1007/BF03041153.

[41] Costoya JA, Hobbs RM, Barna M, Cattoretti G, Manova K et al. (2004) Essential role of Plzf in maintenance of spermatogonial stem cells. Nat Genet 36: 653-659. doi:10.1038/ng1367. PubMed: 15156143. 15156143

[42] Zhang L, Tang J, Haines CJ, Feng HL, Lai L et al. (2011) c-kit and its related genes in spermatogonial differentiation. Spermatogenesis1:186-194. 10.4161/spmg.1.3.17760 PMC3271661 22319667

[43] Dann CT, Alvarado AL, Molyneux LA, Denard BS, Garbers DL et al. (2008) Spermatogonial stem cell self-renewal requires OCT4, a factor downregulated during retinoic acid-induced differentiation. Stem Cells 26: 2928-2937. doi:10.1634/stemcells.2008-0134. PubMed: 18719224. 18719224

[44] Zhou Q, Li Y, Nie R, Friel P, Mitchell D et al. (2008) Expression of Stimulated by Retinoic Acid Gene 8 (Stra8) and Maturation of Murine Gonocytes and Spermatogonia Induced by Retinoic Acid. In Vitro - Biol Reprod 78: 537-545. doi:10.1095/biolreprod.107.064337. 18032419 PMC3208258

[45] Bowles J, Knight D, Smith C, Wilhelm D, Richman J et al. (2006) Retinoid signaling determines germ cell fate in mice. Science 312: 596-600. doi:10.1126/science.1125691. PubMed: 16574820. 16574820

[46] Snyder EM, Davis JC, Zhou Q, Evanoff R, Griswold MD (2011) Exposure to retinoic acid in the neonatal but not adult mouse results in synchronous spermatogenesis. Biol Reprod 84(5): 886-893. doi:10.1095/biolreprod.110.089755. PubMed: 21228214. 21228214 PMC3080418

[47] Hogarth CA, Griswold MD (2010) The key role of vitamin A in spermatogenesis. J Clin Invest 120: 956-962. doi:10.1172/JCI41303. PubMed: 20364093. 20364093 PMC2846058

[48] Verhoeven G, Willems A, Denolet E, Swinnen JV, De Gendt K (2010) Androgens and spermatogenesis: lessons from transgenic mouse models. Philos Trans R Soc Lond B Biol Sci 27: 1537-1556. PubMed: 20403868. 10.1098/rstb.2009.0117 PMC2871915 20403868

[49] Tucci P, Cione E, Genchi G (2008) Retinoic acid-induced testosterone production and retinoylation reaction are concomitant and exhibit a positive correlation in Leydig (TM-3) cells. J Bioenerg Biomembr 40: 111-115. doi:10.1007/s10863-008-9132-3. PubMed: 18324454. 18324454

[50] Chung SS, Choi C, Wang X, Hallock L, Wolgemuth DJ (2010) Aberrant distribution of junctional complex components in retinoic acid receptor alpha-deficient mice. Microsc Res Tech 73: 583-596. PubMed: 19937743. 19937743 10.1002/jemt.20797 PMC2877760

[51] Gohbara A, Katagiri K, Sato T, Kubota Y, Kagechika H et al. (2010) In vitro murine spermatogenesis in an organ culture system. Biol Reprod 83: 261-267. doi:10.1095/biolreprod.110.083899. PubMed: 20393168. 20393168

[52] Sato T, Katagiri K, Gohbara A, Inoue K, Ogonuki N et al. (2011) In vitro production of functional sperm in cultured neonatal mouse testes. Nature 471: 504-508. doi:10.1038/nature09850. PubMed: 21430778. 21430778

[53] Lee JH, Kim HJ, Kim H, Lee SJ, Gye MC (2006) In vitro spermatogenesis by three-dimensional culture of rat testicular cells in collagen gel matrix. Biomaterials 27: 2845-2853. doi:10.1016/j.biomaterials.2005.12.028. PubMed: 16430959. 16430959

[54] Lee JH, Gye MC, Choi KW, Hong JY, Lee YB et al. (2007) In vitro differentiation of germ cells from nonobstructive azoospermic patients using three-dimensional culture in a collagen gel matrix. Fertil Steril 87: 824-833. doi:10.1016/j.fertnstert.2006.09.015. PubMed: 17239867. 17239867

[55] Stukenborg JB, Wistuba J, Luetjens CM, Elhija MA, Huleihel M et al. (2008) Coculture of spermatogonia with somatic cells in a novel three-dimensional soft-agar-culture-system. J Androl 29: 312-329. doi:10.2164/jandrol.107.002857. PubMed: 18046051. 18046051

[56] Stukenborg JB, Schlatt S, Simoni M, Yeung CH, Elhija MA et al. (2009) New horizons for in vitro spermatogenesis? An update on novel three-dimensional culture systems as tools for meiotic and post-meiotic differentiation of testicular germ cells. Mol Hum Reprod 15: 521-529. doi:10.1093/molehr/gap052. PubMed: 19561342. 19561342

[57] Vernet N, Dennefeld C, Rochette-Egly C, Oulad-Abdelghani M, Chambon P et al. (2006) Retinoic acid metabolism and signaling pathways in the adult and developing mouse testis. Endocrinology 147: 96-110. PubMed: 16210368. 16210368 10.1210/en.2005-0953

[58] Bowles J, Koopman P (2007) Retinoic acid, meiosis and germ cell fate in mammals. Development 134: 3401-3411. doi:10.1242/dev.001107. PubMed: 17715177. 17715177

[59] Woods EJ, Benson JD, Agca Y, Critser JK (2004) Fundamental cryobiology of reproductive cells and tissues. Cryobiology 48: 146-156. doi:10.1016/j.cryobiol.2004.03.002. PubMed: 15094091. 15094091

[60] Curaba M, Verleysen M, Amorim CA, Dolmans MM, Van Langendonckt A et al. (2011) Cryopreservation of prepubertal mouse testicular tissue by vitrification. Fertil Steril 15: 1229-1234. PubMed: 20541745. 10.1016/j.fertnstert.2010.04.062 20541745

[61] Keros V, Rosenlund B, Hultenby K, Aghajanova L, Levkov L et al. (2005) Optimizing cryopreservation of human testicular tissue: comparison of protocols with glycerol, propanediol and dimethylsulphoxide as cryoprotectants. Hum Reprod 20: 1676-1687. doi:10.1093/humrep/deh797. PubMed: 15860503. 15860503

[62] Kvist K, Thorup J, Byskov AG, Høyer PE, Møllgård K et al. (2006) Cryopreservation of intact testicular tissue from boys with cryptorchidism. Hum Reprod 21: 484-491. PubMed: 16210383. 16210383 10.1093/humrep/dei331

