# Supplementary figures and images for: Effects of Vitamin A on In Vitro Maturation of Pre-Pubertal Mouse Spermatogonial Stem Cells
Source: PLoS One. 2013 Dec 9;8(12):e82819. doi: 10.1371/journal.pone.0082819 (PMC3857286; doi:10.1371/journal.pone.0082819)

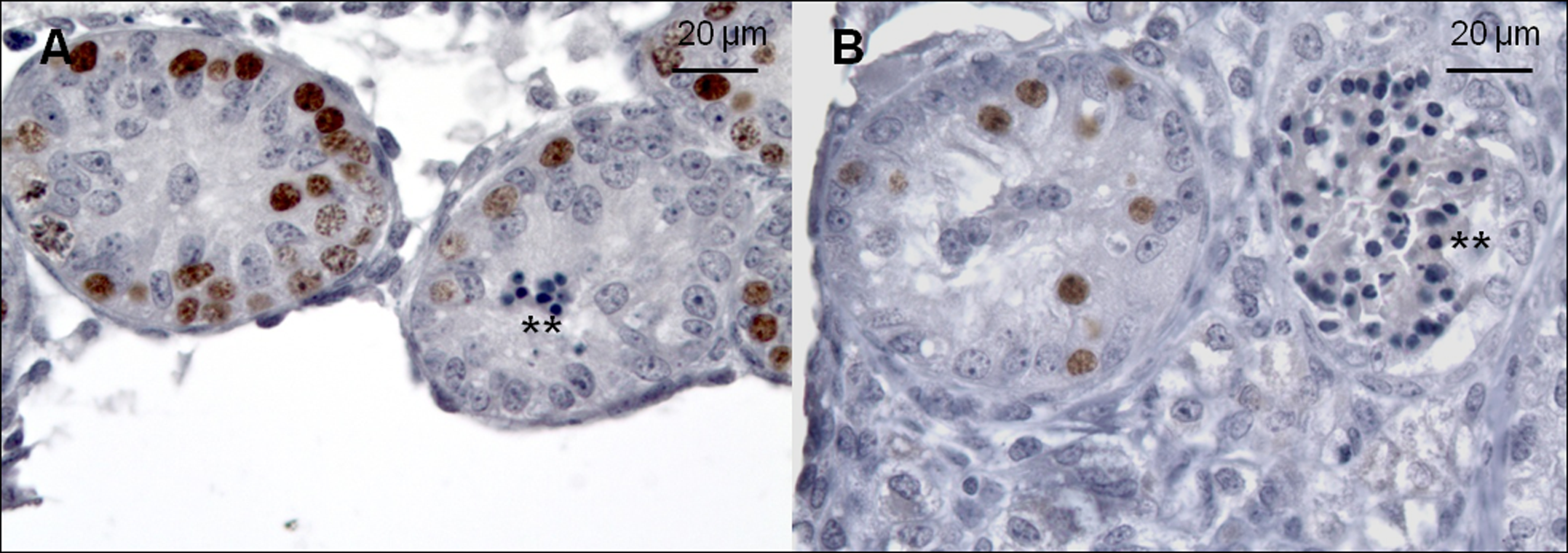

Supplement: Figure S1 — Histological evaluation using Tra98 immunostaining of seminiferous tubules obtained after organotypic culture of pre-pubertal mice testes at days 7 (A) and days 11 (B) of culture using a culture medium containing 10-5M retinoic acid. Photomicrographs were captured at ×500 magnification. Intra-tubular cells with pycnotic nuclei (black asterisks) were not stained with Tra98 antibody. The distinction between pycnotic Sertoli cells and pycnotic spermatogonia was not possible. (TIF) [file pone.0082819.s001.tif]

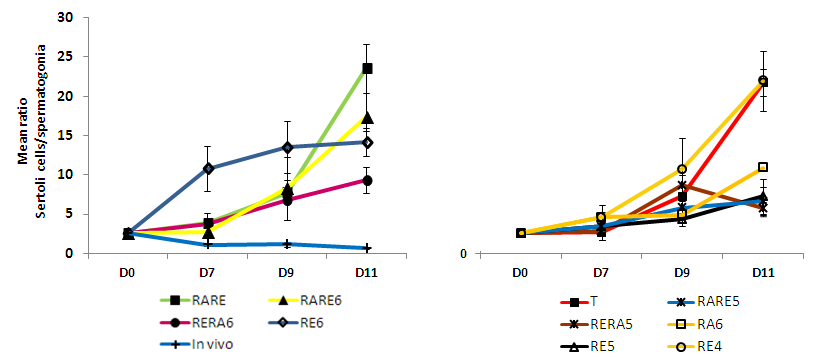

Supplement: Figure S2 — Ratio between Sertoli cells and spermatogonia in organotypic culture of fresh pre-pubertal mouse testicular tissue. The evolution of the ratio between Sertoli cells and spermatogonia was assessed after 0, 7, 9 and 11 days of organotypic culture on at least 30 cross-sectioned tubules after Tra98 immunostaining under the different culture conditions. The results are represented as the mean ± SEM with n=6. Footnotes: T: basal culture medium without retinoid; RARE: 3.3.10-7M RA and 3.3.10-7M RE; RARE6: 3.3.10-7M RA and 10-6M RE; RARE5: 3.3.10-7M RA and 10-5M RE; RERA6: 3.3.10-7M RE and 10-6M RA; RERA5: 3.3.10-7M RE and 10-5M RA; RA6: 10-6M RA; RE6: 10-6M RE; RE5: 10-5M RE; RE4: 10-4M RE; RE3: 10-3M RE; In vivo: In vivo control. RA: Retinoic acid; RE: Retinol; D0: Day 0; D7: Day 7; D9: Day 9; D11: Day 11 (TIF) [file pone.0082819.s002.tif]

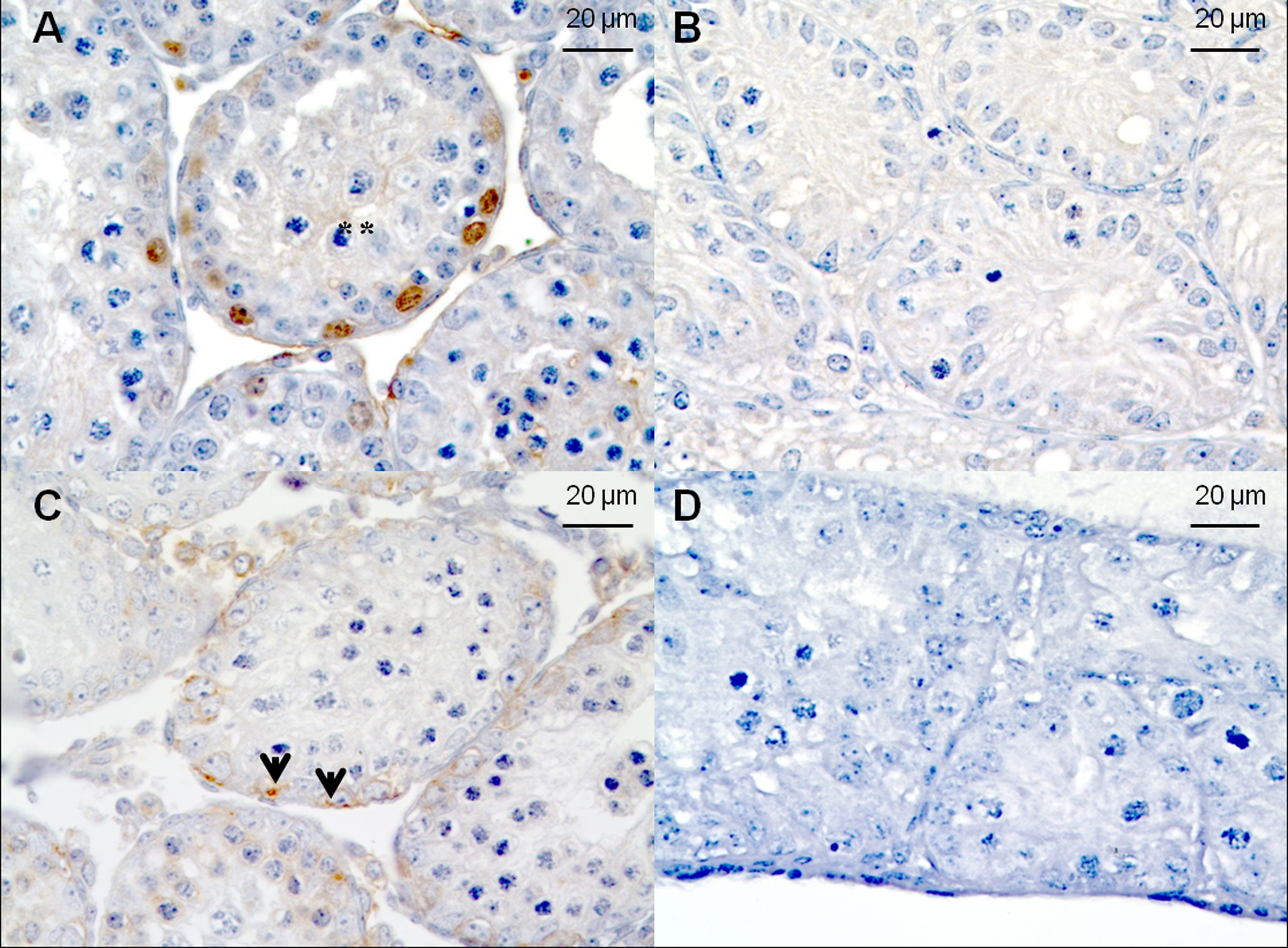

Supplement: Figure S3 — Immunohistochemistry with an antibody to Promyelocytic leukemia zinc finger (Plzf) (A-B) and an antibody to c-kit (C-D) on testicular tissue sections from 14 days post-partum (dpp) old mice and from organotypic culture at days 9 of culture using a culture medium containing 10-6M retinol. Photomicrographs were captured at ×500 magnification. Brown stained undifferentiated (black asterisks) and differentiated (black arrows) spermatogonia were observed in seminiferous tubules of 14 dpp old mice and were not detected after organotypic culture of testicular tissue of pre-pubertal mice testes. (TIF) [file pone.0082819.s003.tif]

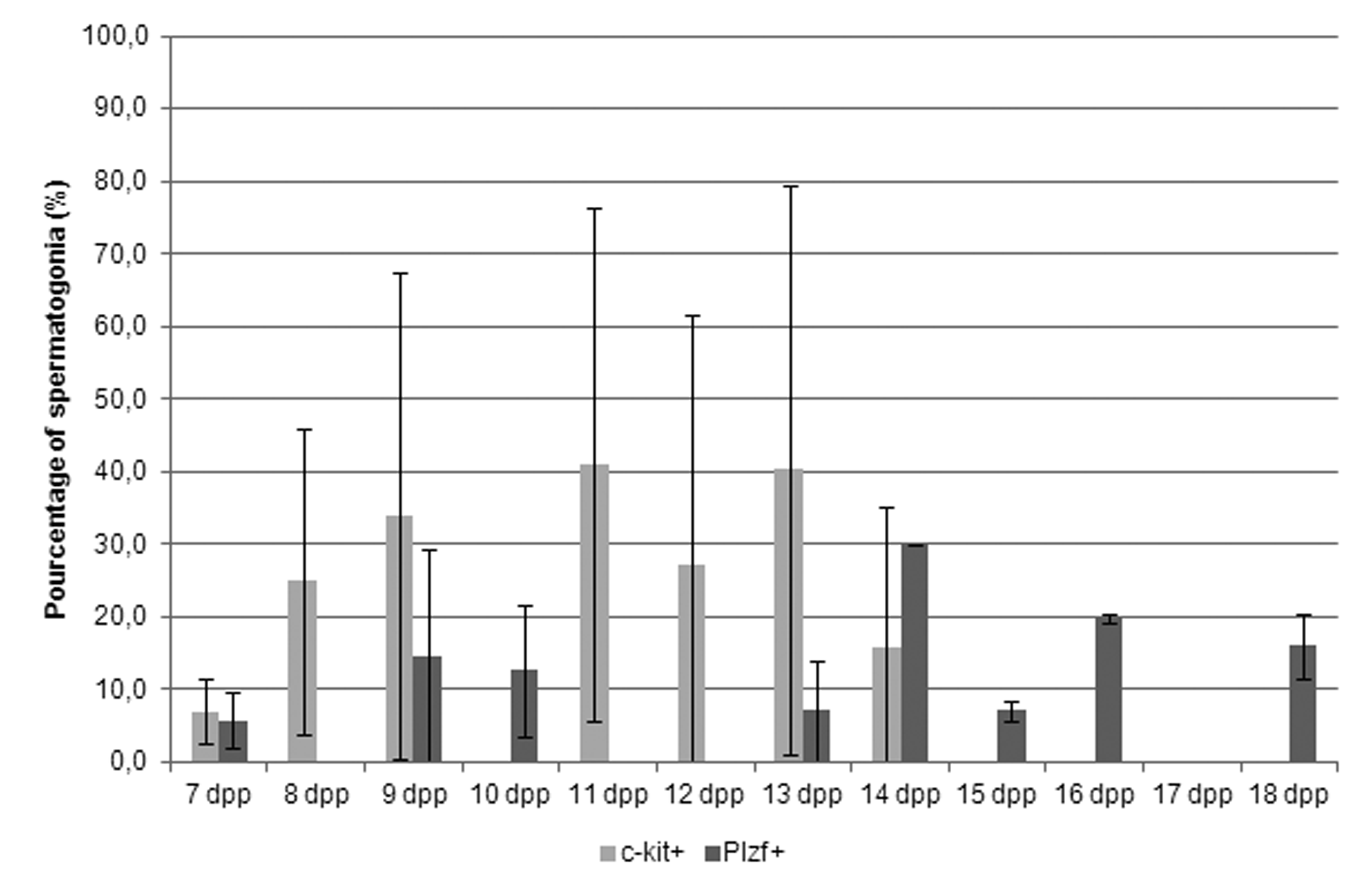

Supplement: Figure S4 — Assessment of Promyelocytic leukemia zinc finger (Plzf) and c-kit expression in spermatogonia of mice seminiferous tubules from 7 days post partum (dpp) to 18 dpp. The results are presented as the mean±SEM with n=2. (TIF) [file pone.0082819.s004.tif]

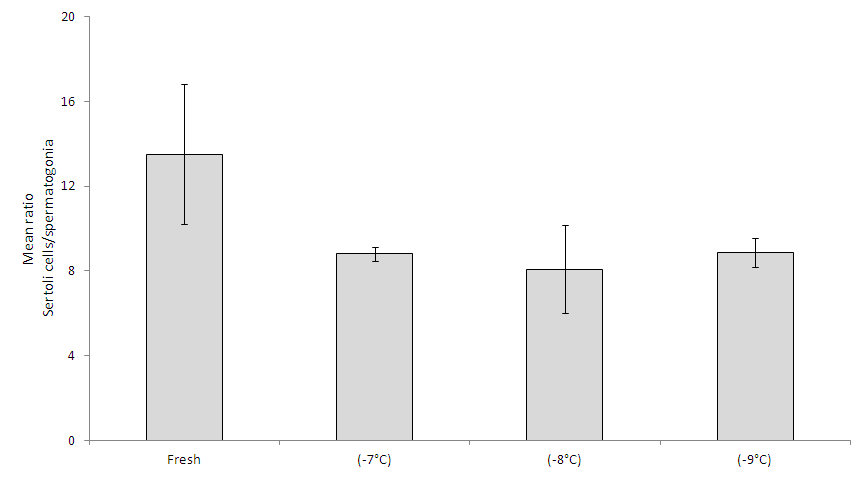

Supplement: Figure S5 — Ratio between Sertoli cells and spermatogonia of frozen-thawed pre-pubertal mouse testicular tissue after 9 days of culture with 10-6M retinol. Results were compared with fresh pre-pubertal testicular tissue cultured with the same conditions. Testicular tissue was cryopreserved using a controlled slow freezing protocol and a soaking temperature evaluated at -7°C, -8°C or -9°C. (TIF) [file pone.0082819.s005.tif]
